# Supplementary figures and images for: Herpetin Promotes Bone Marrow Mesenchymal Stem Cells to Alleviate Carbon Tetrachloride-Induced Acute Liver Injury in Mice
Source: Molecules. 2023 May 1;28(9):3842. doi: 10.3390/molecules28093842 (PMC10180416; doi:10.3390/molecules28093842)

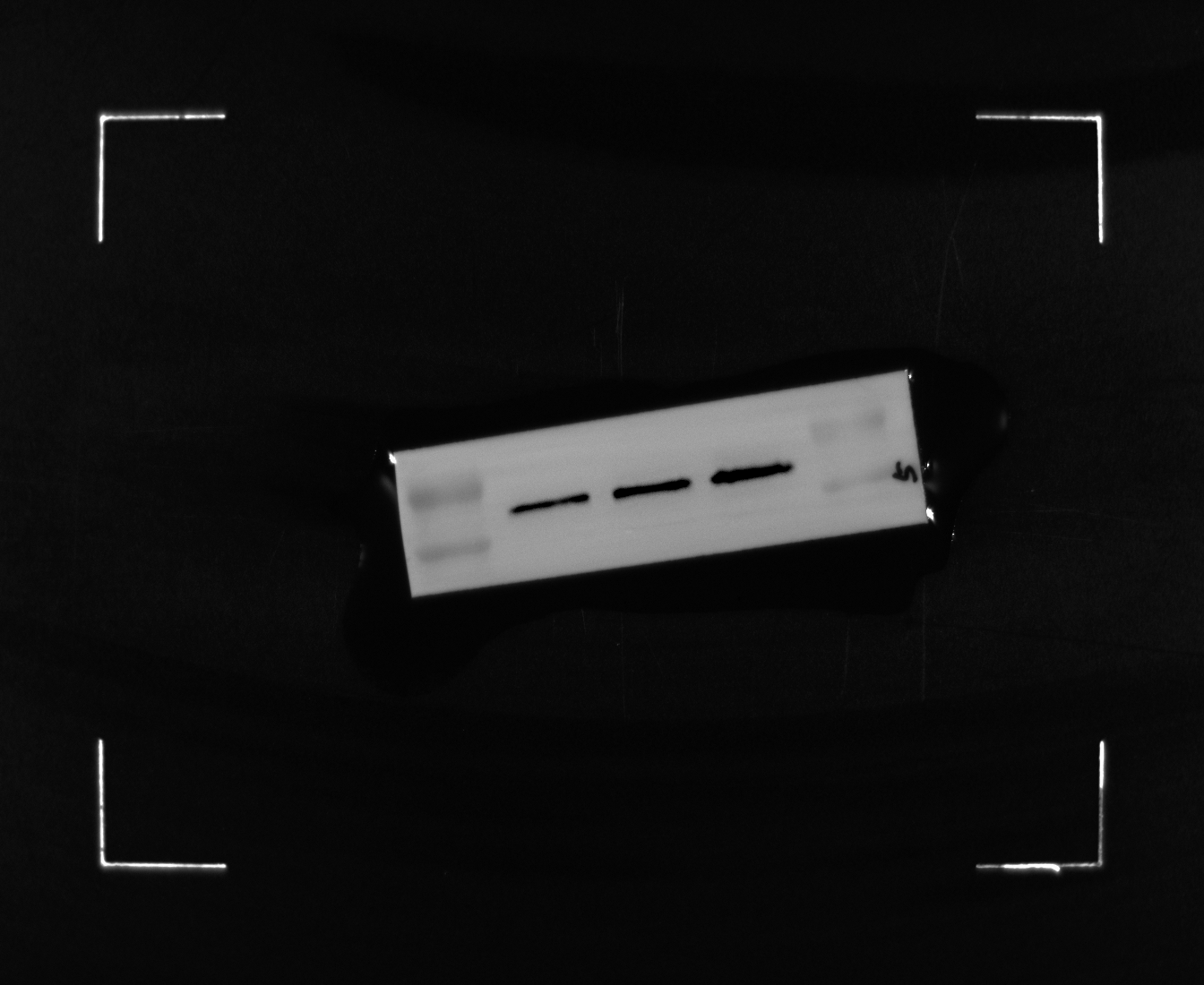

Supplement: Supplementary file 1 [file molecules-28-03842-s001.zip › WB original picture/AFP ALB CK18/14d/AFP-5.tif]

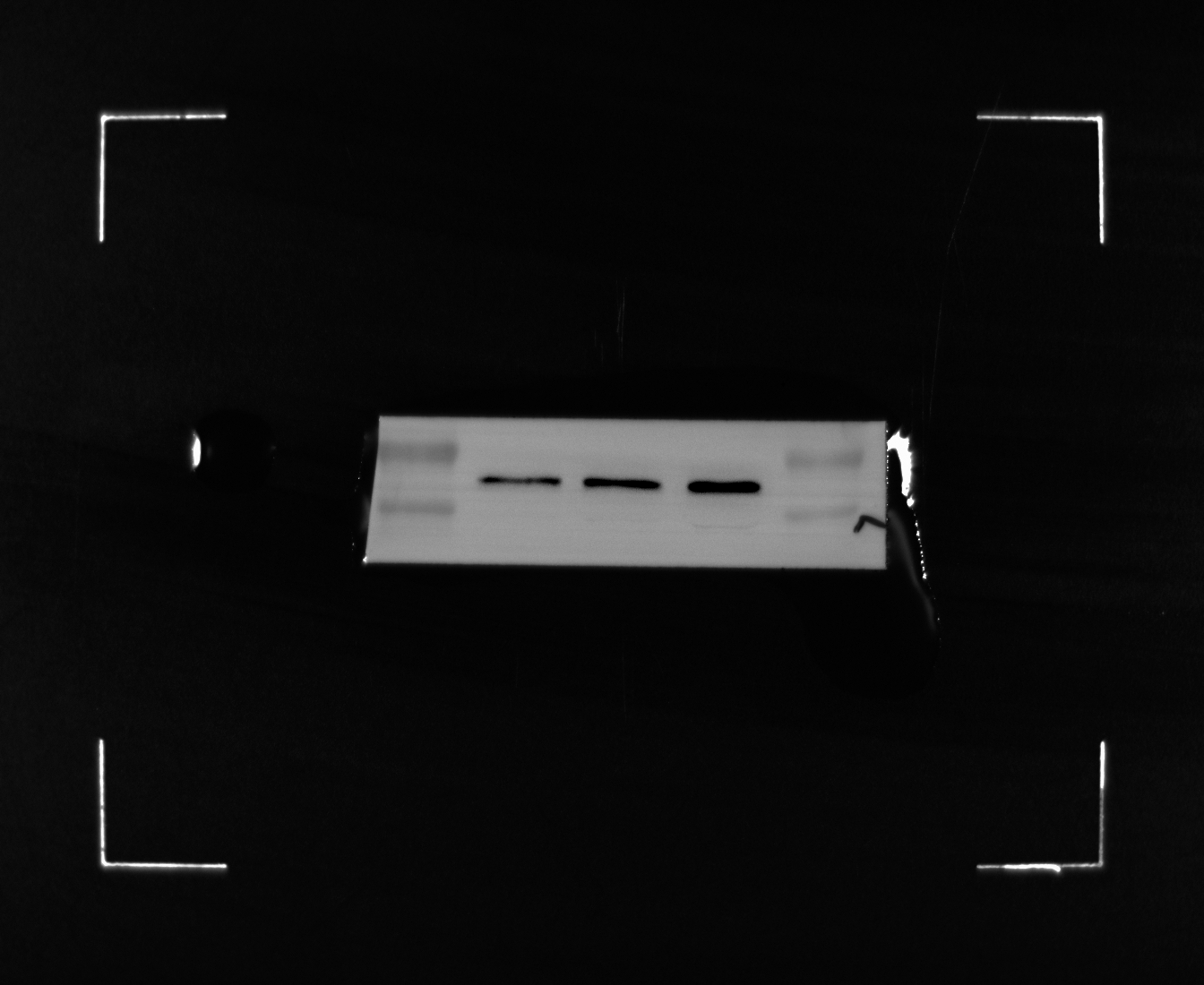

Supplement: Supplementary file 1 [file molecules-28-03842-s001.zip › WB original picture/AFP ALB CK18/14d/AFP-7.tif]

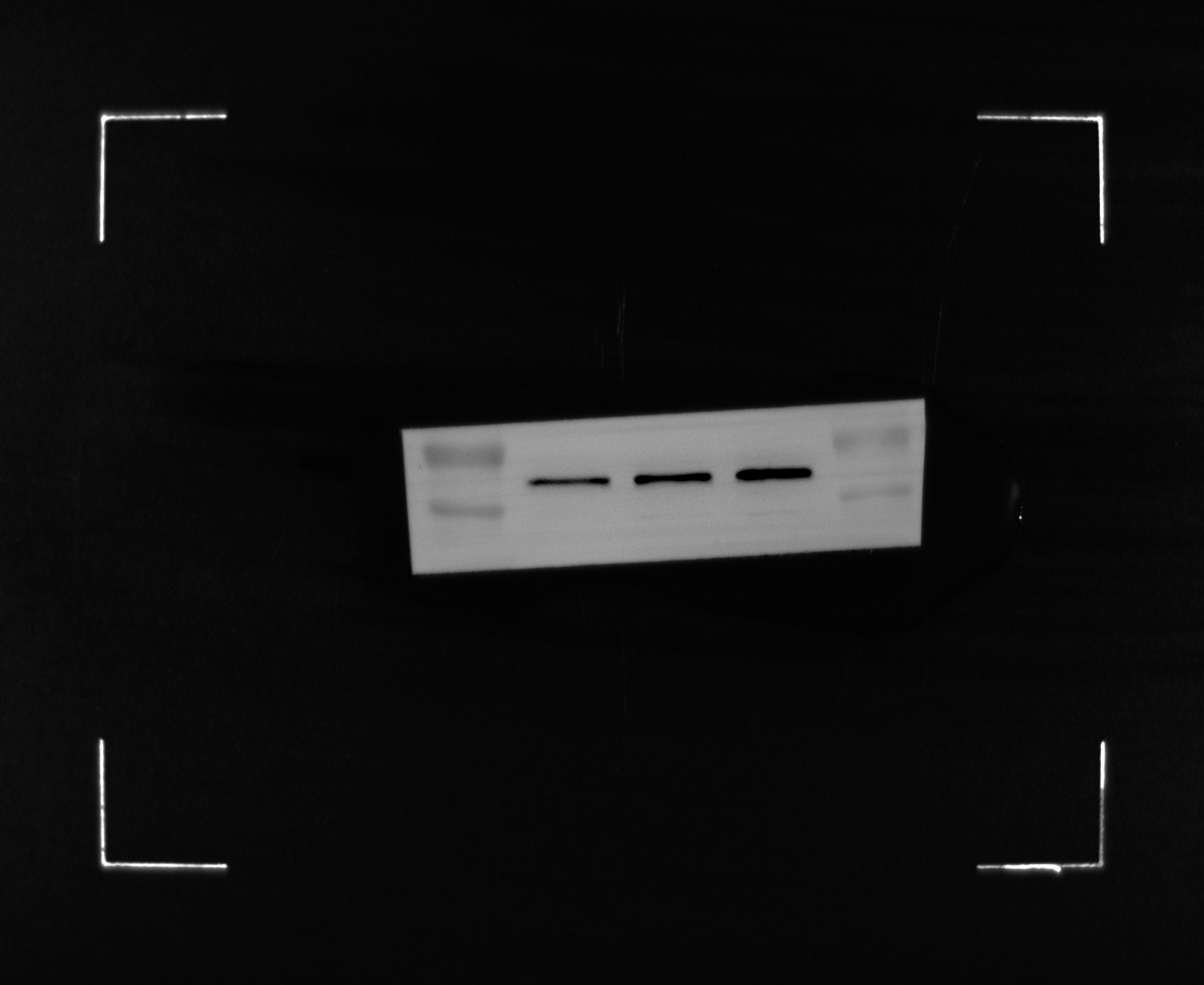

Supplement: Supplementary file 1 [file molecules-28-03842-s001.zip › WB original picture/AFP ALB CK18/14d/AFP-8.tif]

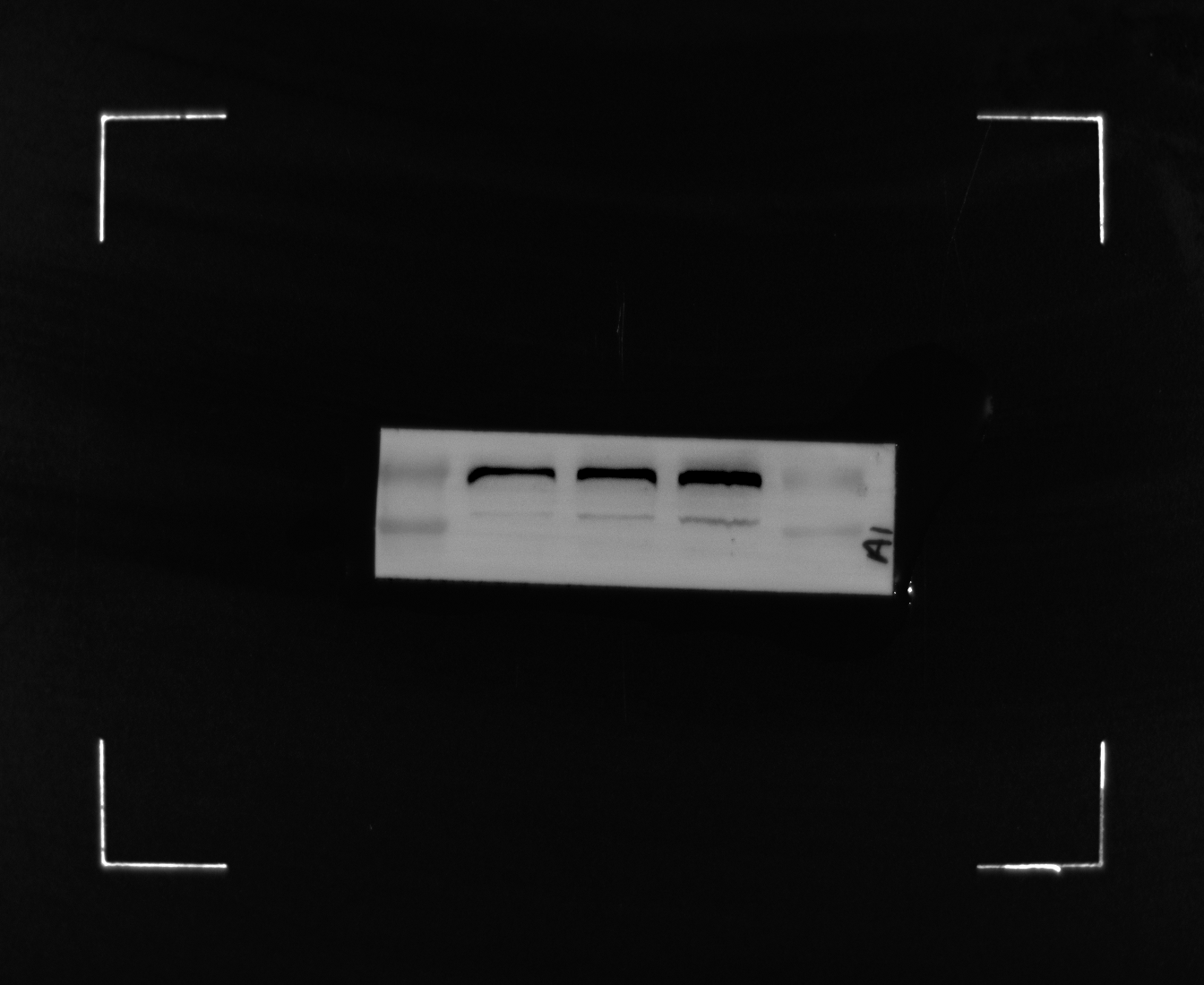

Supplement: Supplementary file 1 [file molecules-28-03842-s001.zip › WB original picture/AFP ALB CK18/14d/ALB.tif]

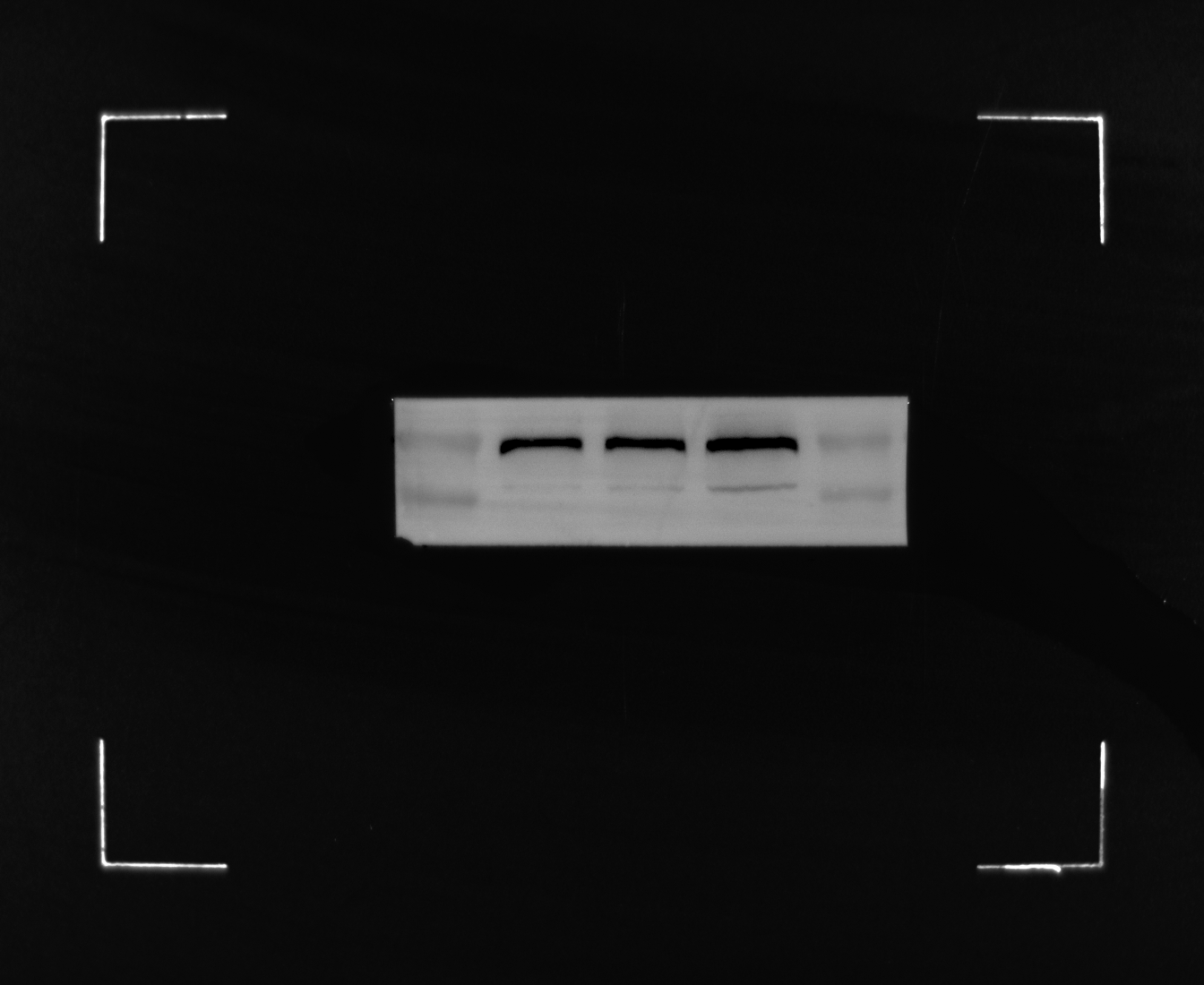

Supplement: Supplementary file 1 [file molecules-28-03842-s001.zip › WB original picture/AFP ALB CK18/14d/ALB-2.tif]

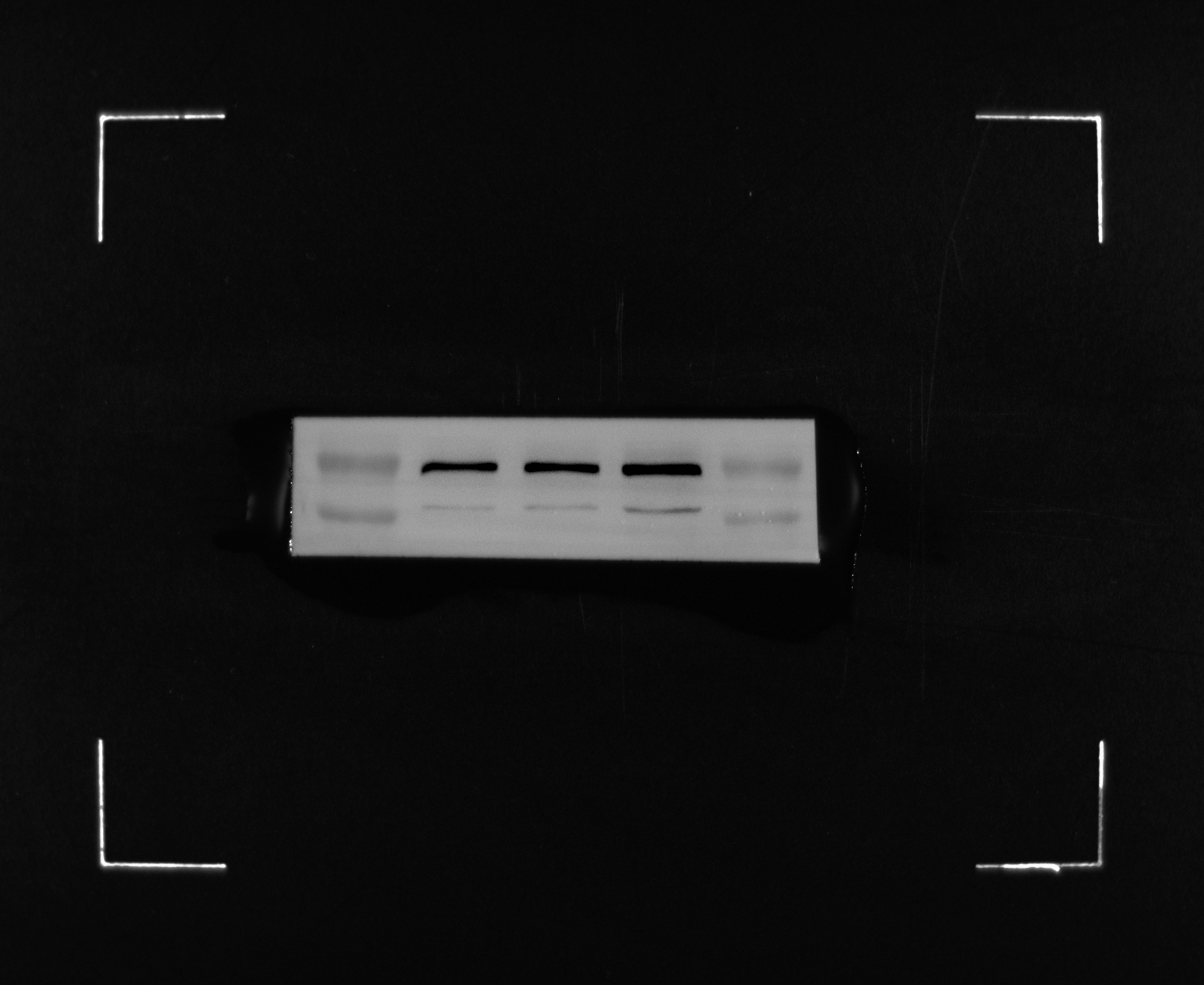

Supplement: Supplementary file 1 [file molecules-28-03842-s001.zip › WB original picture/AFP ALB CK18/14d/ALB-3.tif]

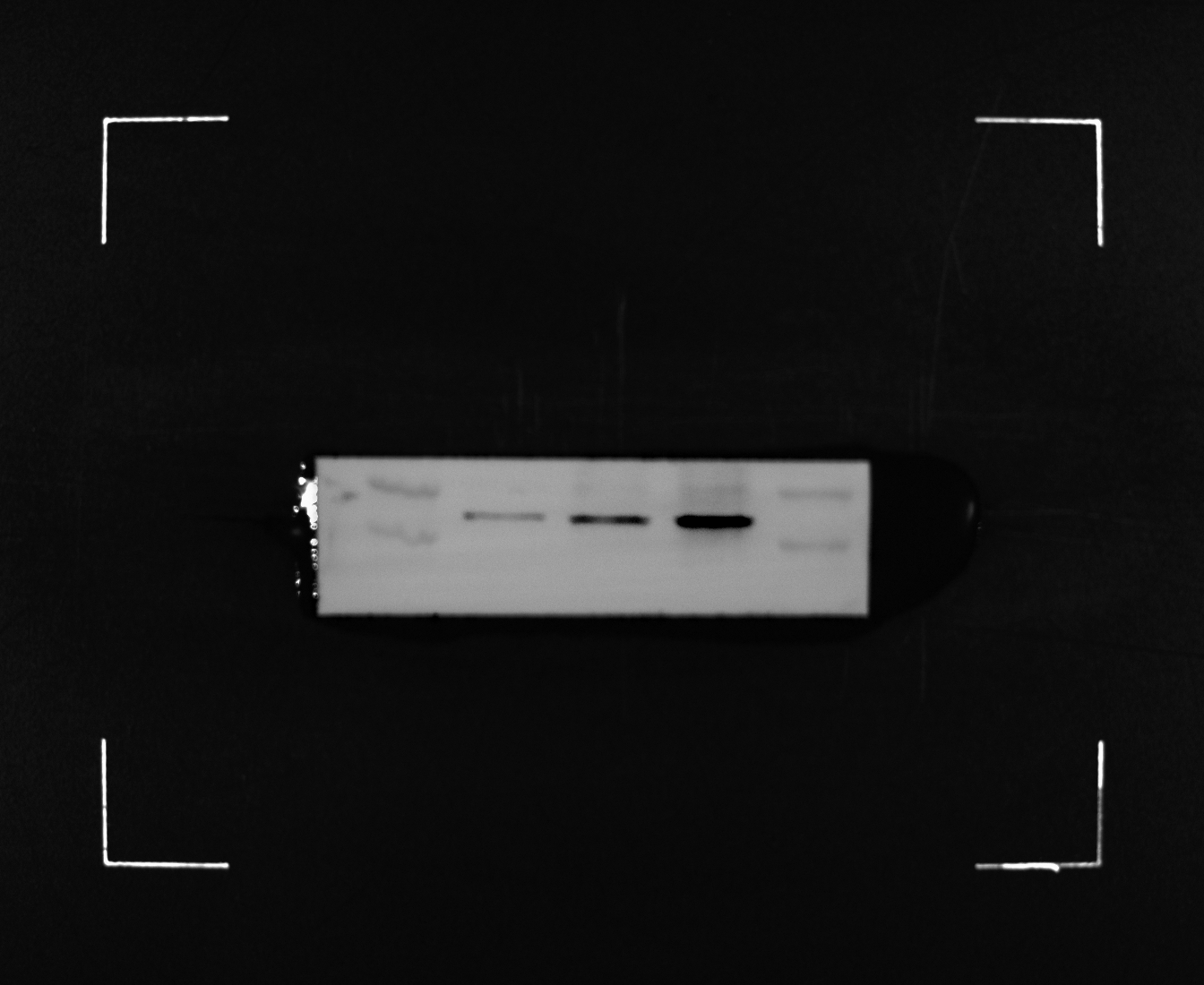

Supplement: Supplementary file 1 [file molecules-28-03842-s001.zip › WB original picture/AFP ALB CK18/14d/CK18.tif]

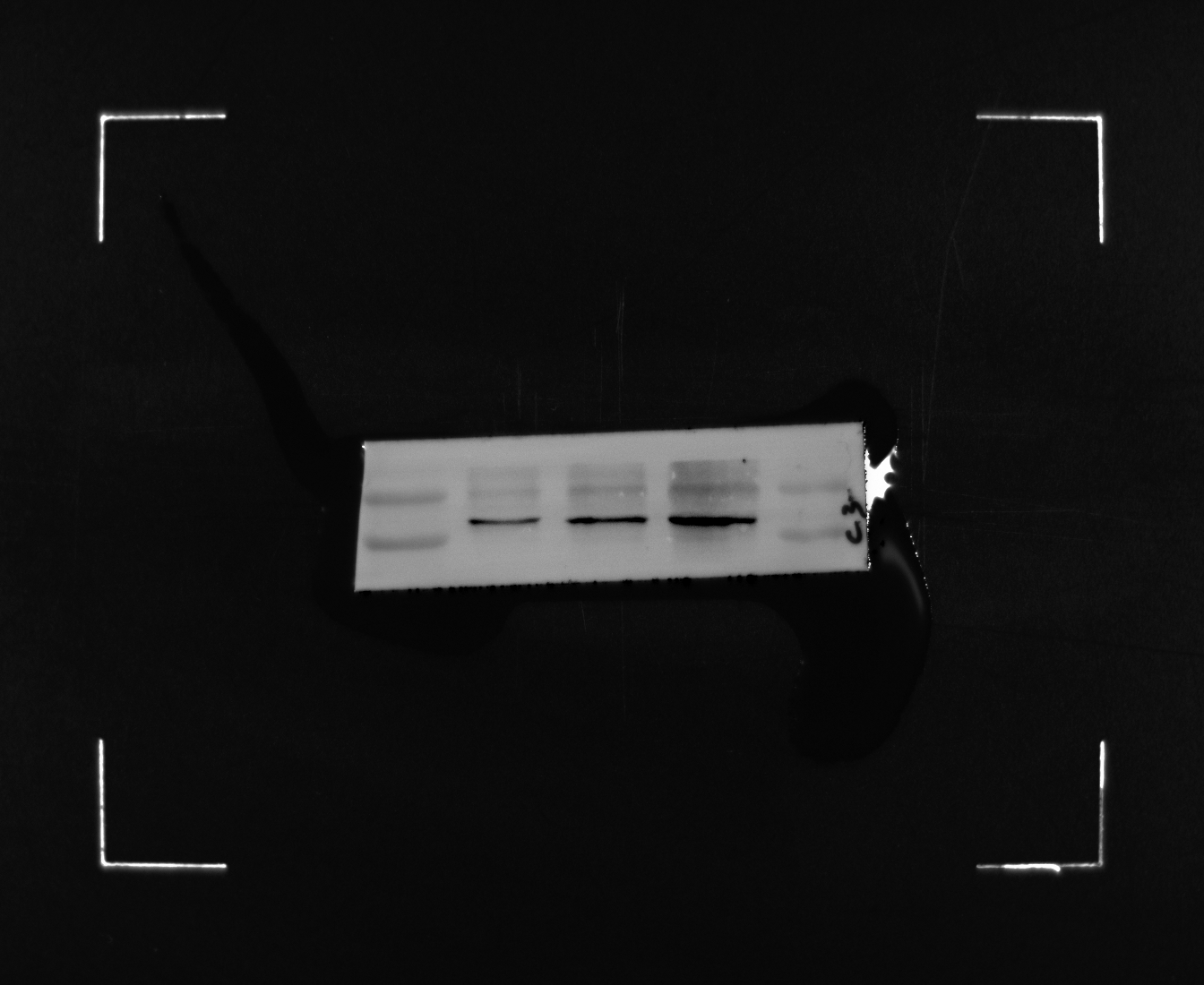

Supplement: Supplementary file 1 [file molecules-28-03842-s001.zip › WB original picture/AFP ALB CK18/14d/CK18-3.tif]

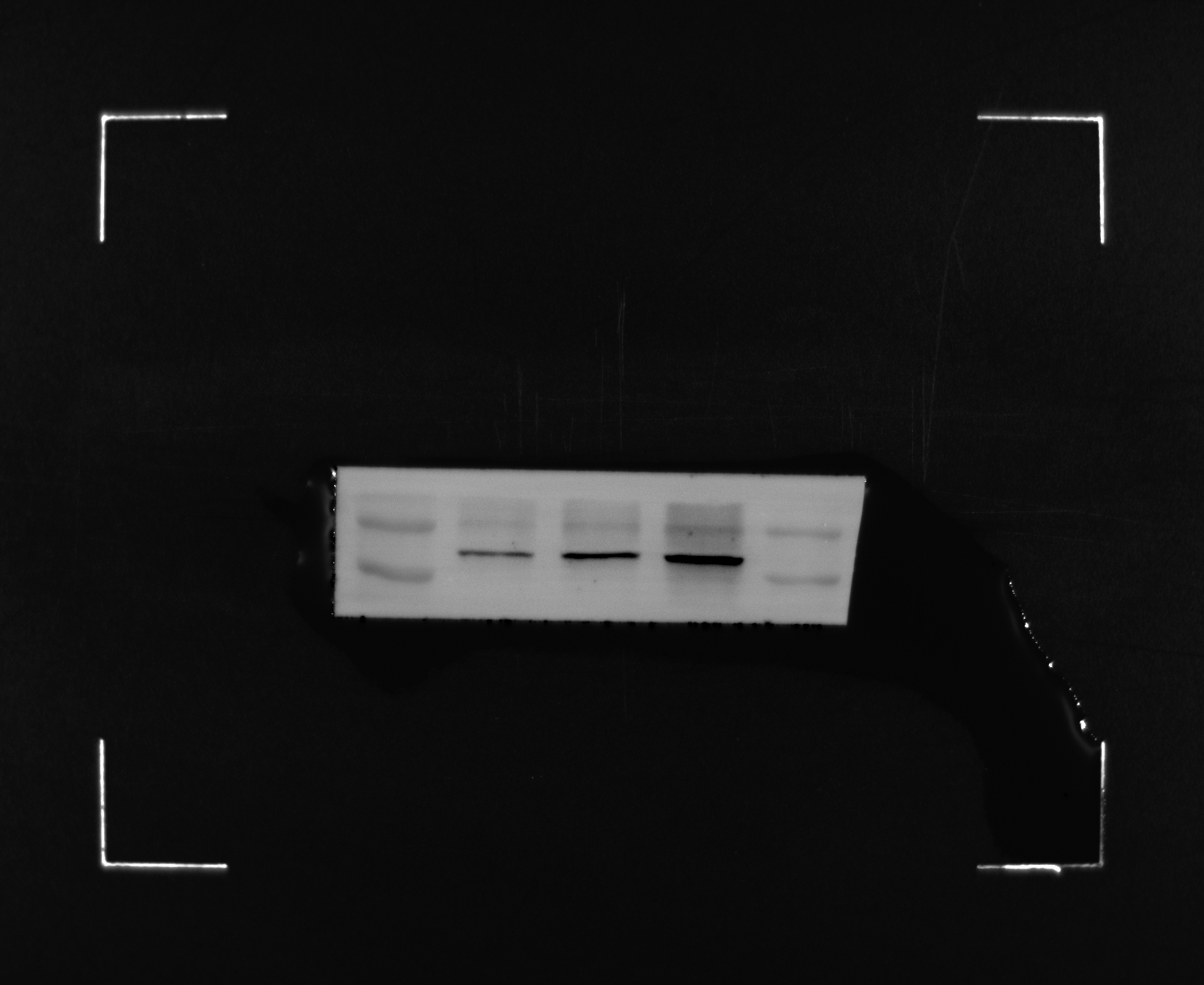

Supplement: Supplementary file 1 [file molecules-28-03842-s001.zip › WB original picture/AFP ALB CK18/14d/CK18-4.tif]

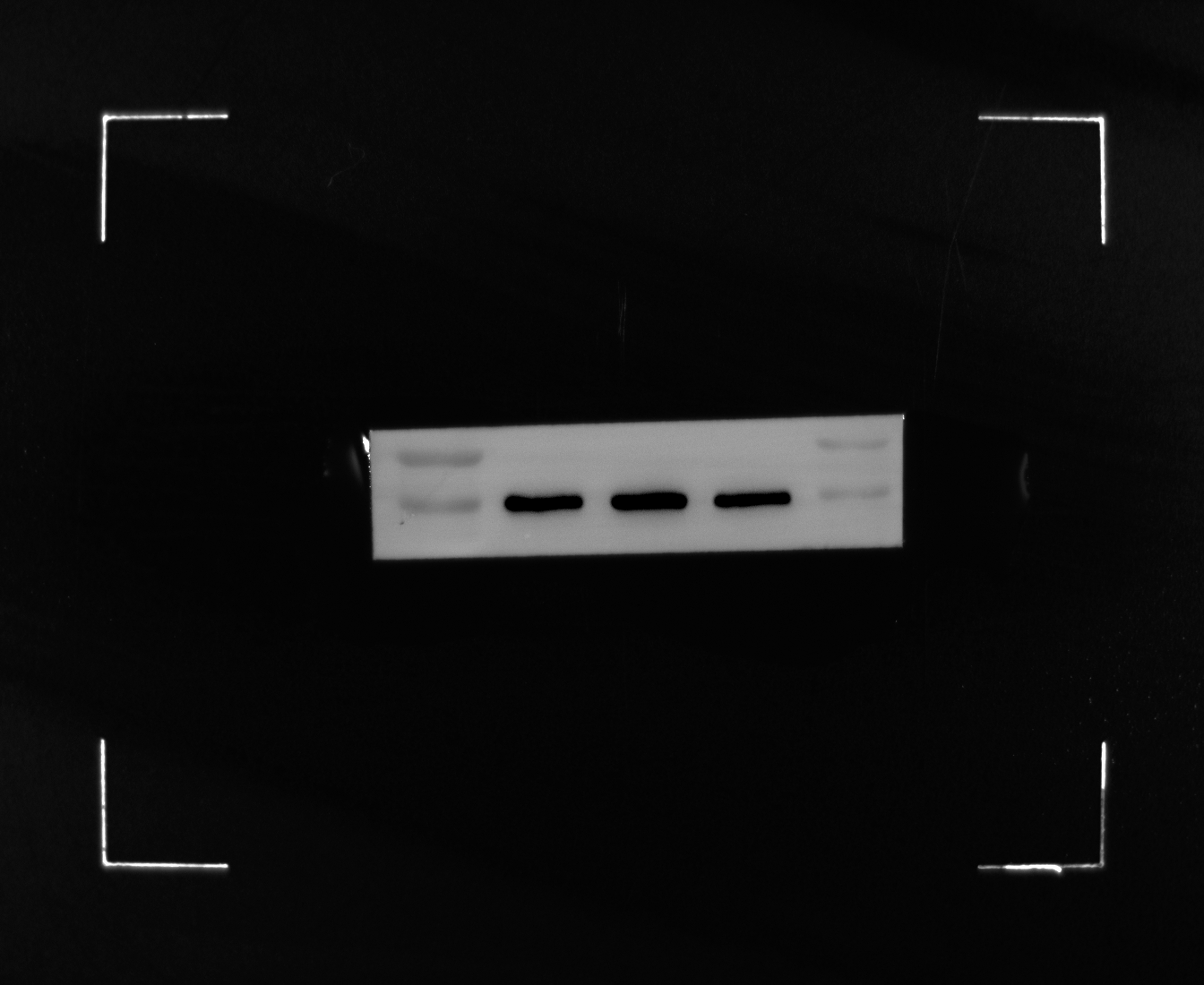

Supplement: Supplementary file 1 [file molecules-28-03842-s001.zip › WB original picture/AFP ALB CK18/14d/GAPDH-4.tif]

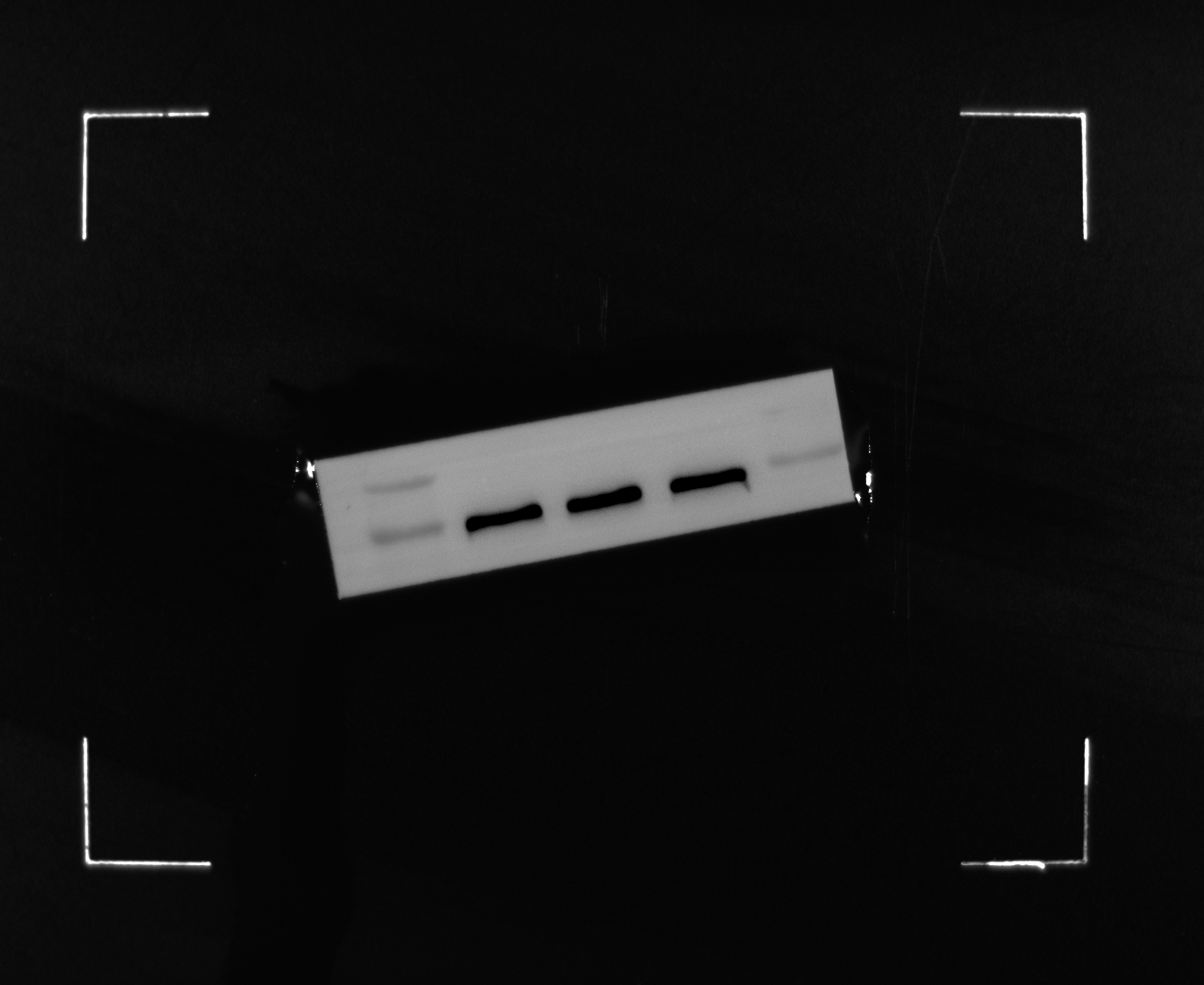

Supplement: Supplementary file 1 [file molecules-28-03842-s001.zip › WB original picture/AFP ALB CK18/14d/GAPDH-6.tif]

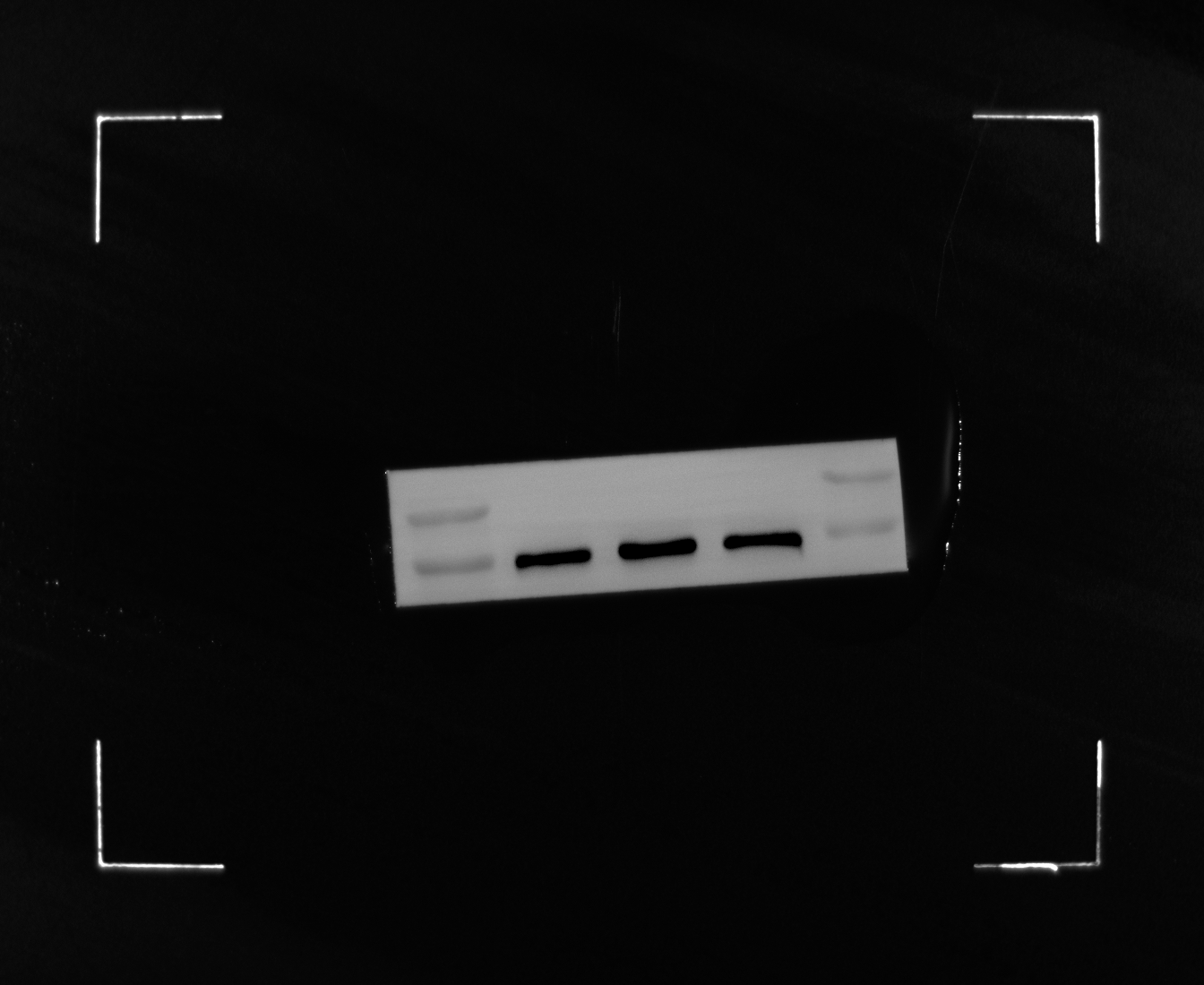

Supplement: Supplementary file 1 [file molecules-28-03842-s001.zip › WB original picture/AFP ALB CK18/14d/GAPDH-8.tif]

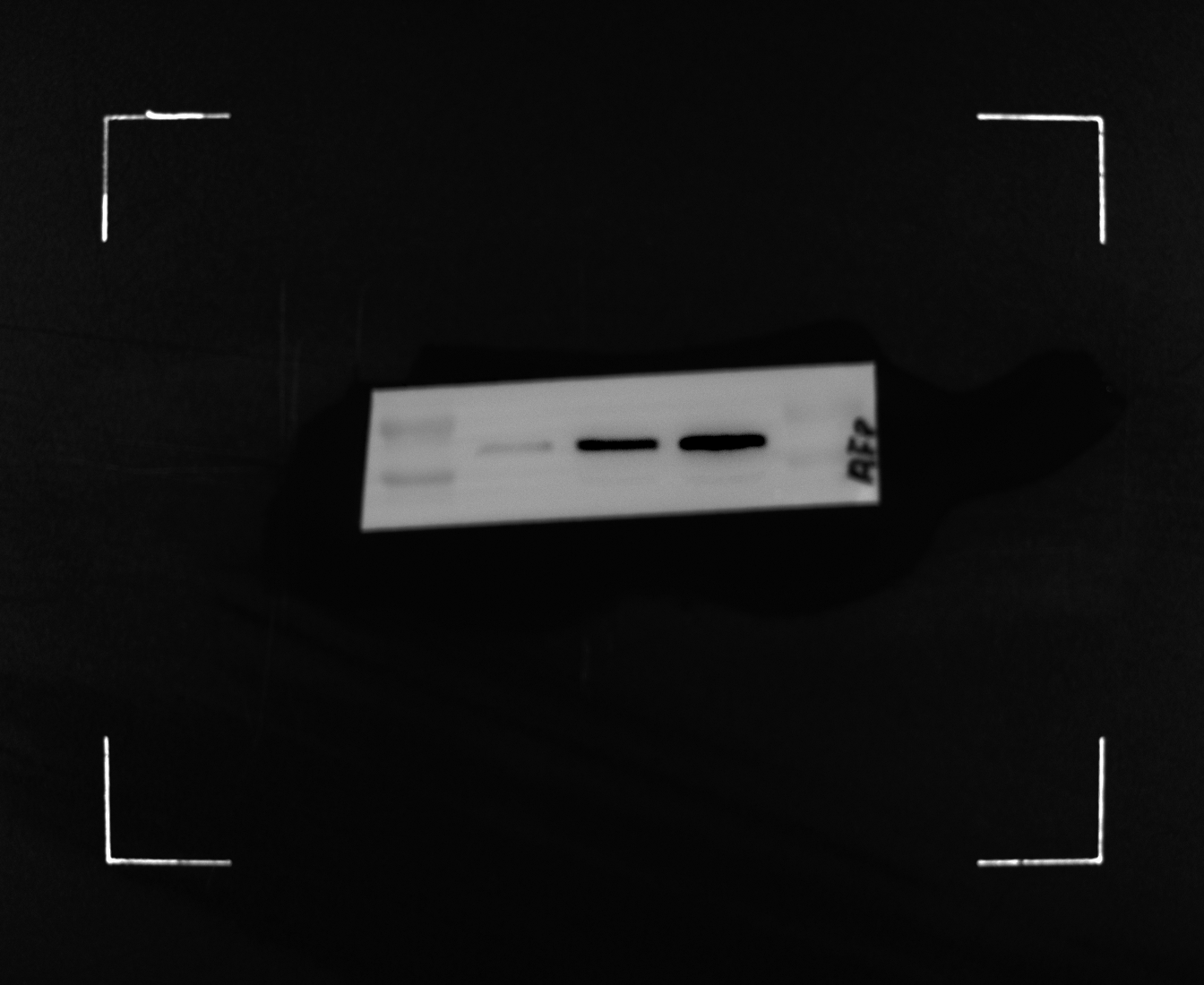

Supplement: Supplementary file 1 [file molecules-28-03842-s001.zip › WB original picture/AFP ALB CK18/21d/AFP.tif]

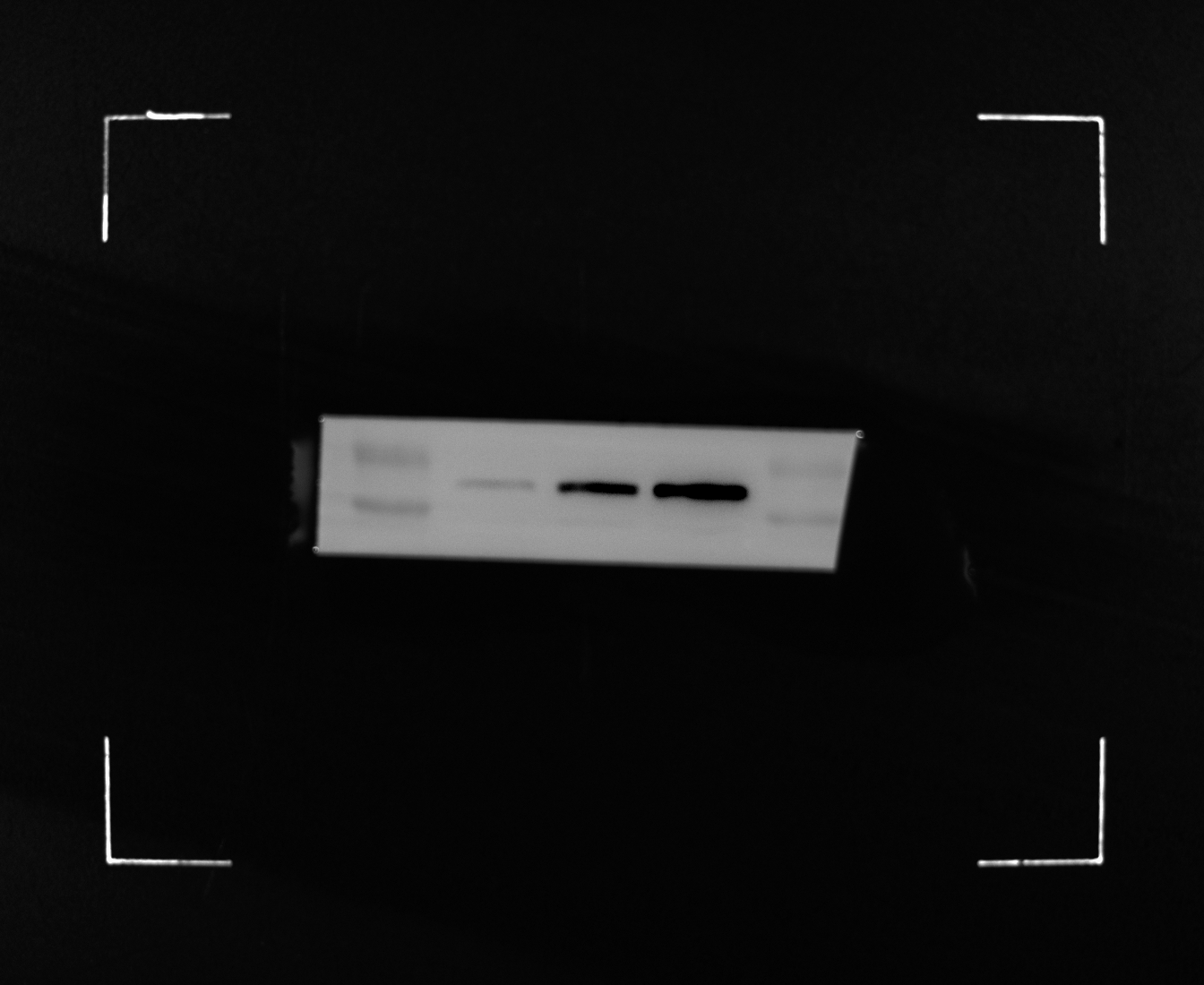

Supplement: Supplementary file 1 [file molecules-28-03842-s001.zip › WB original picture/AFP ALB CK18/21d/AFP-1.tif]

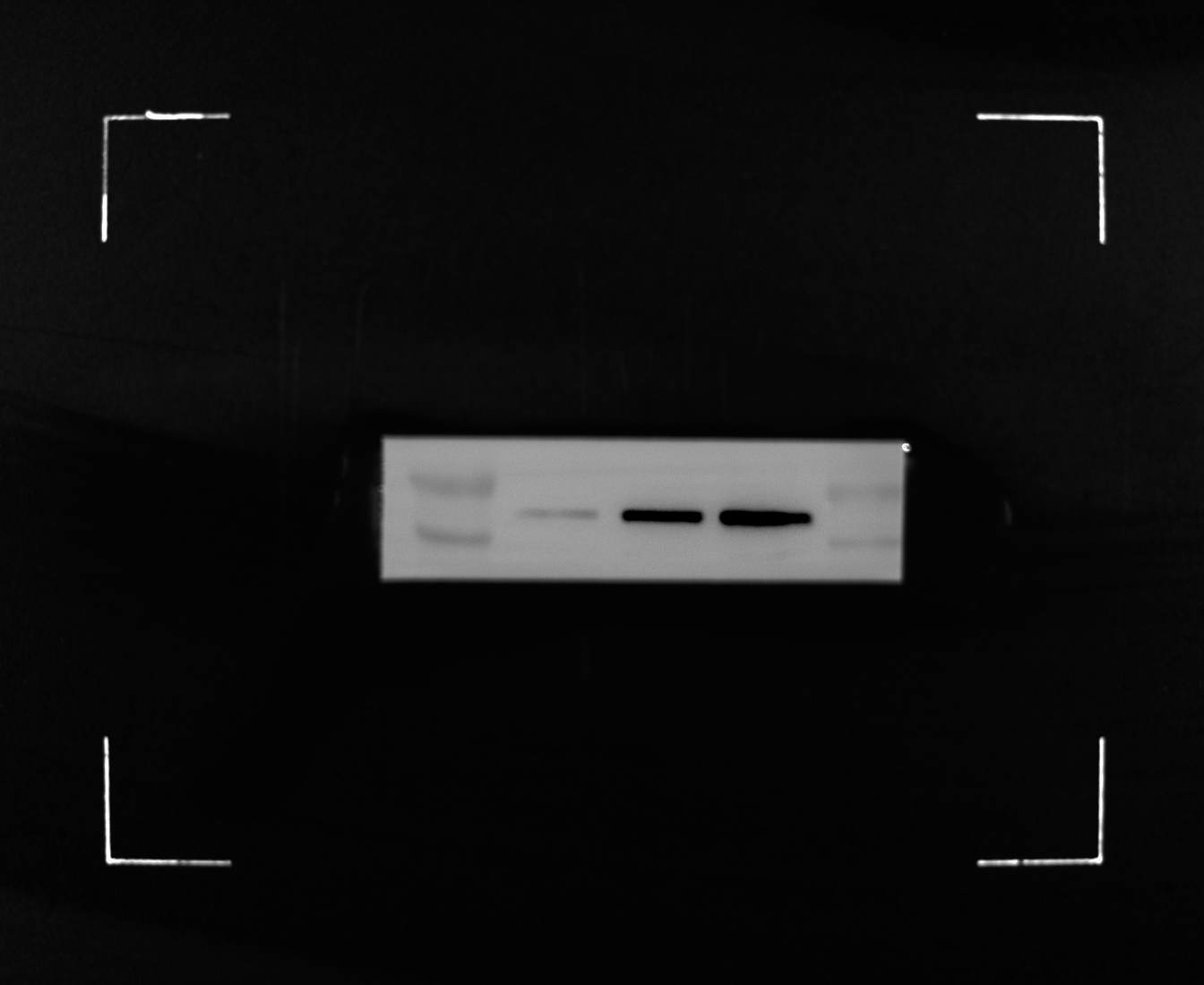

Supplement: Supplementary file 1 [file molecules-28-03842-s001.zip › WB original picture/AFP ALB CK18/21d/AFP-3.tif]

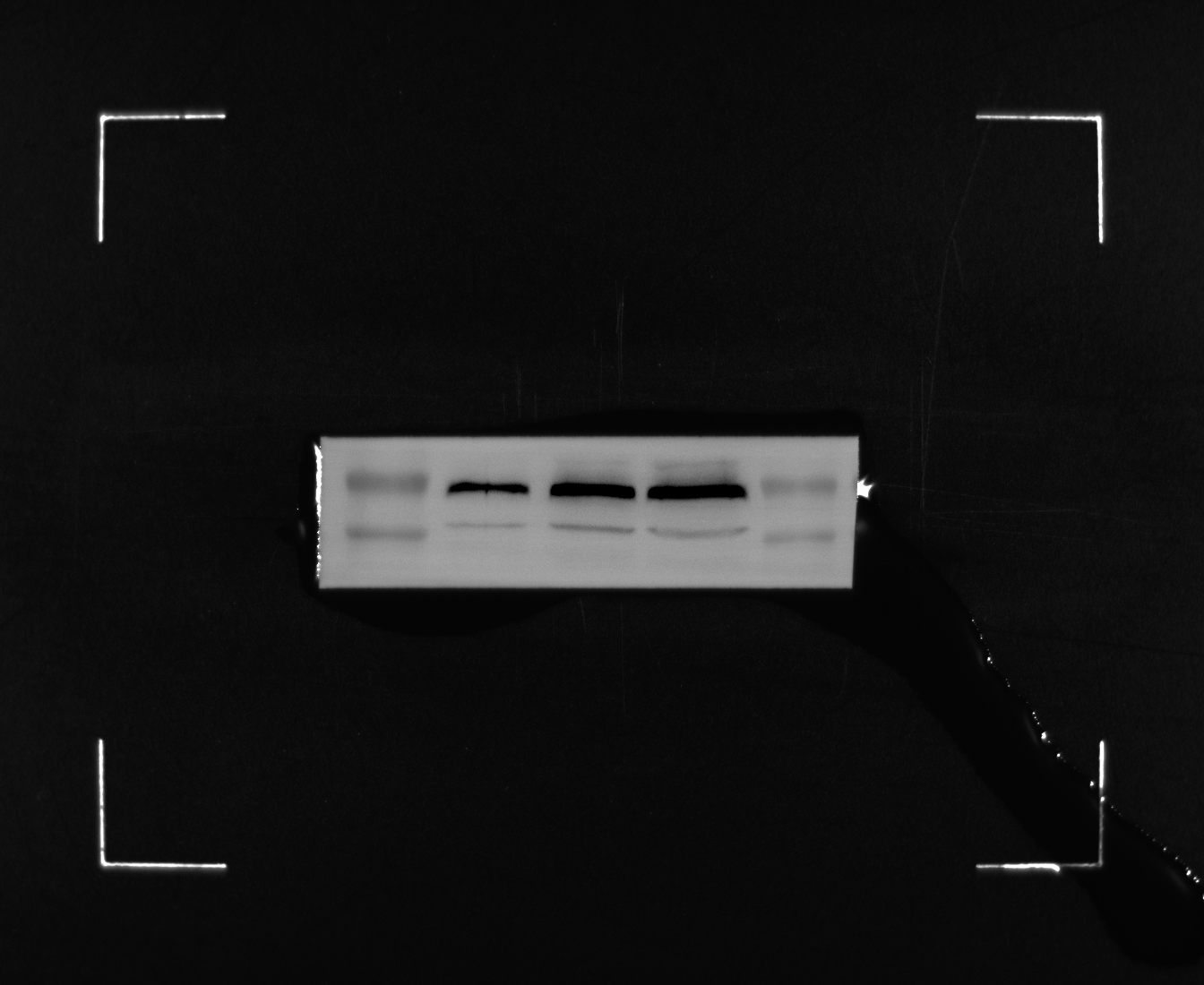

Supplement: Supplementary file 1 [file molecules-28-03842-s001.zip › WB original picture/AFP ALB CK18/21d/ALB.tif]

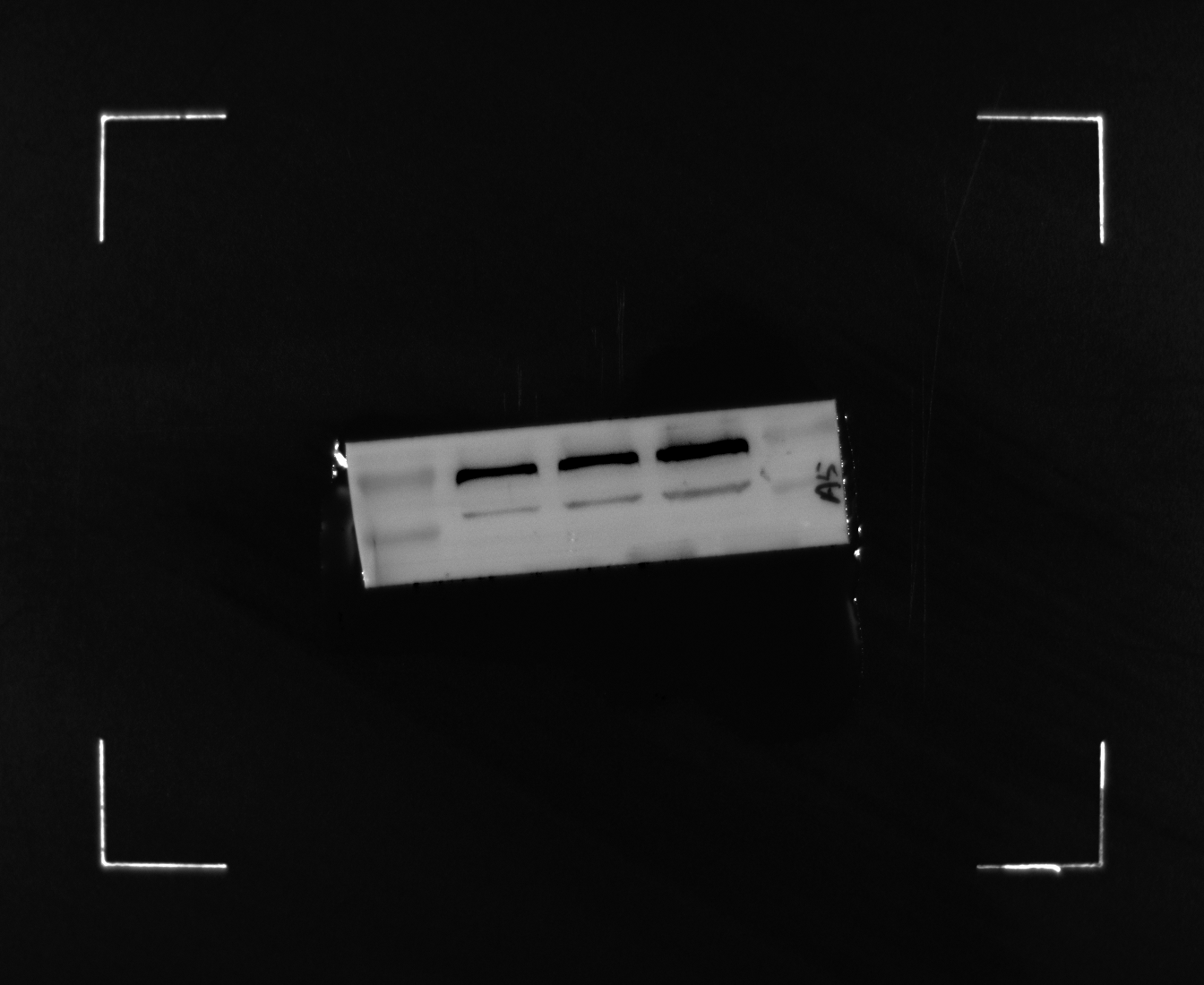

Supplement: Supplementary file 1 [file molecules-28-03842-s001.zip › WB original picture/AFP ALB CK18/21d/ALB-5.tif]

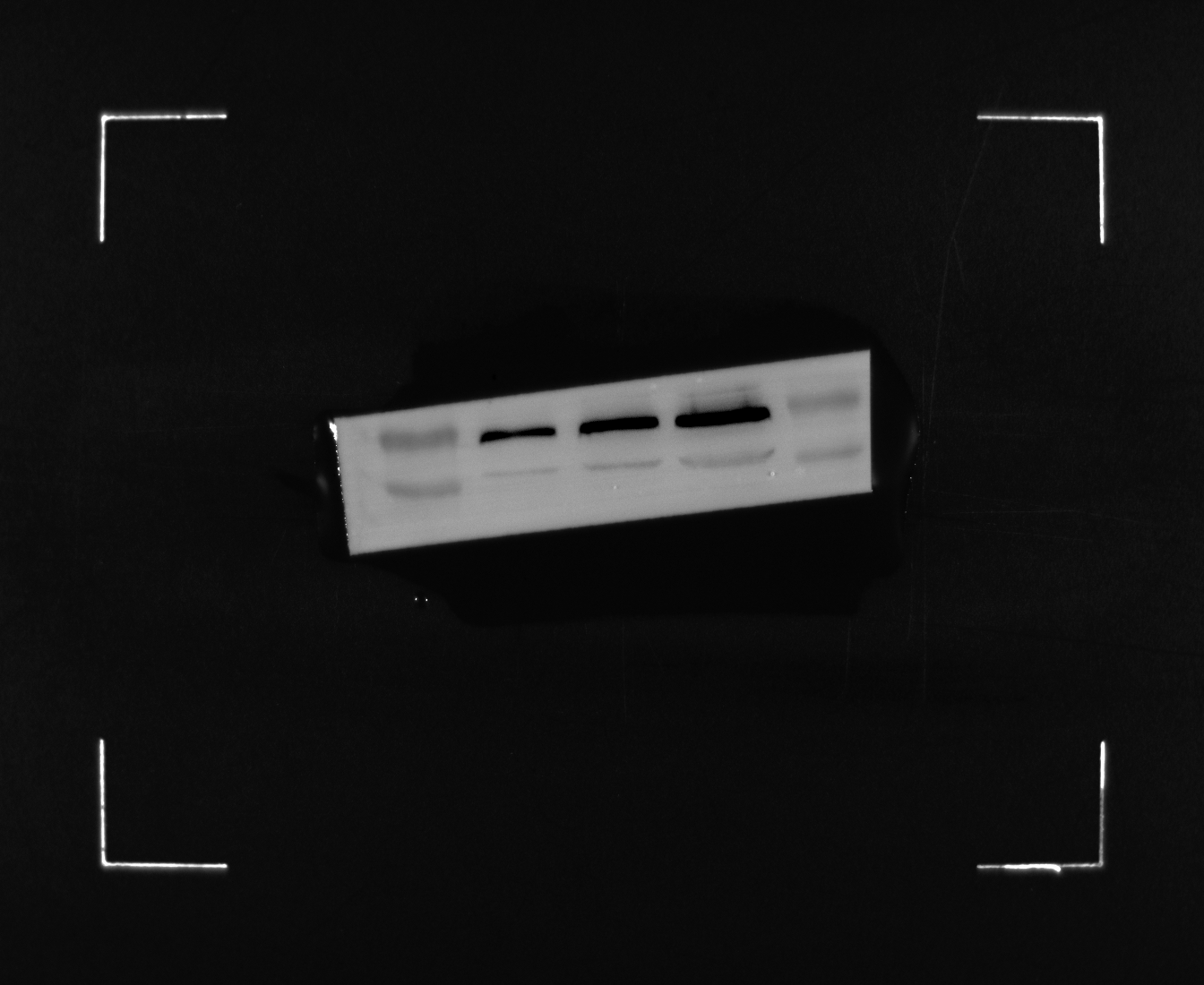

Supplement: Supplementary file 1 [file molecules-28-03842-s001.zip › WB original picture/AFP ALB CK18/21d/ALB-7.tif]

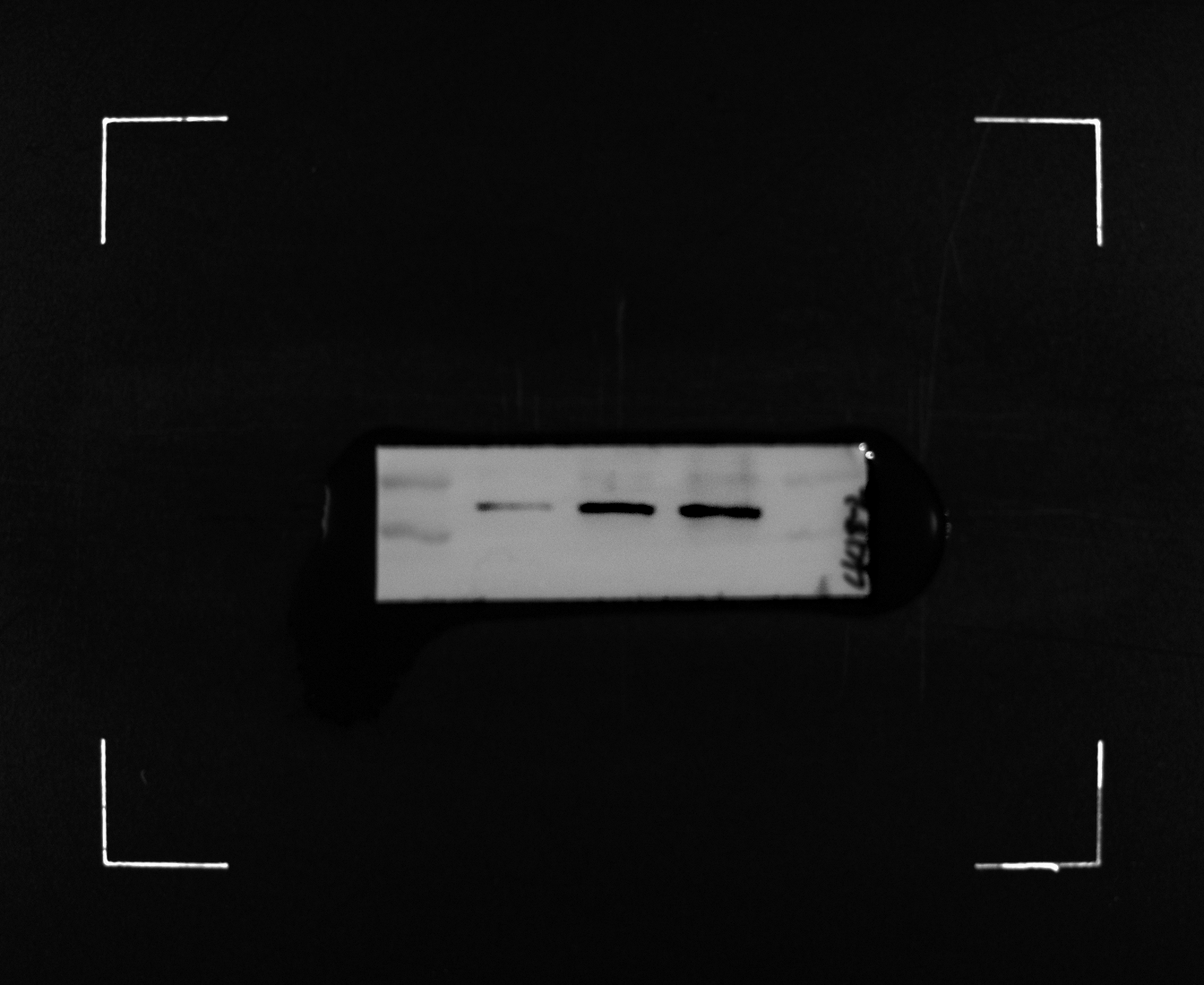

Supplement: Supplementary file 1 [file molecules-28-03842-s001.zip › WB original picture/AFP ALB CK18/21d/CK18-2.tif]

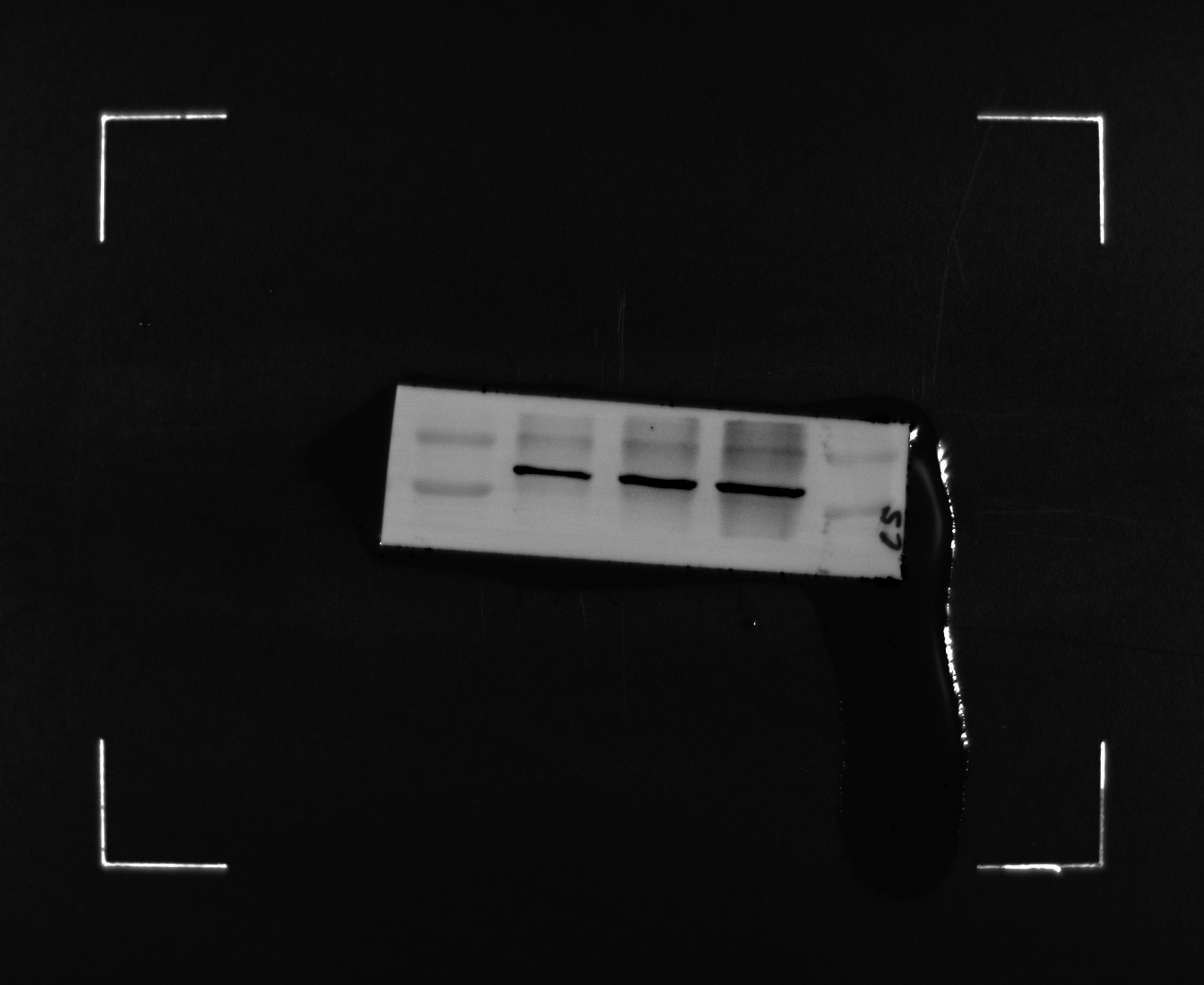

Supplement: Supplementary file 1 [file molecules-28-03842-s001.zip › WB original picture/AFP ALB CK18/21d/CK18-5.tif]

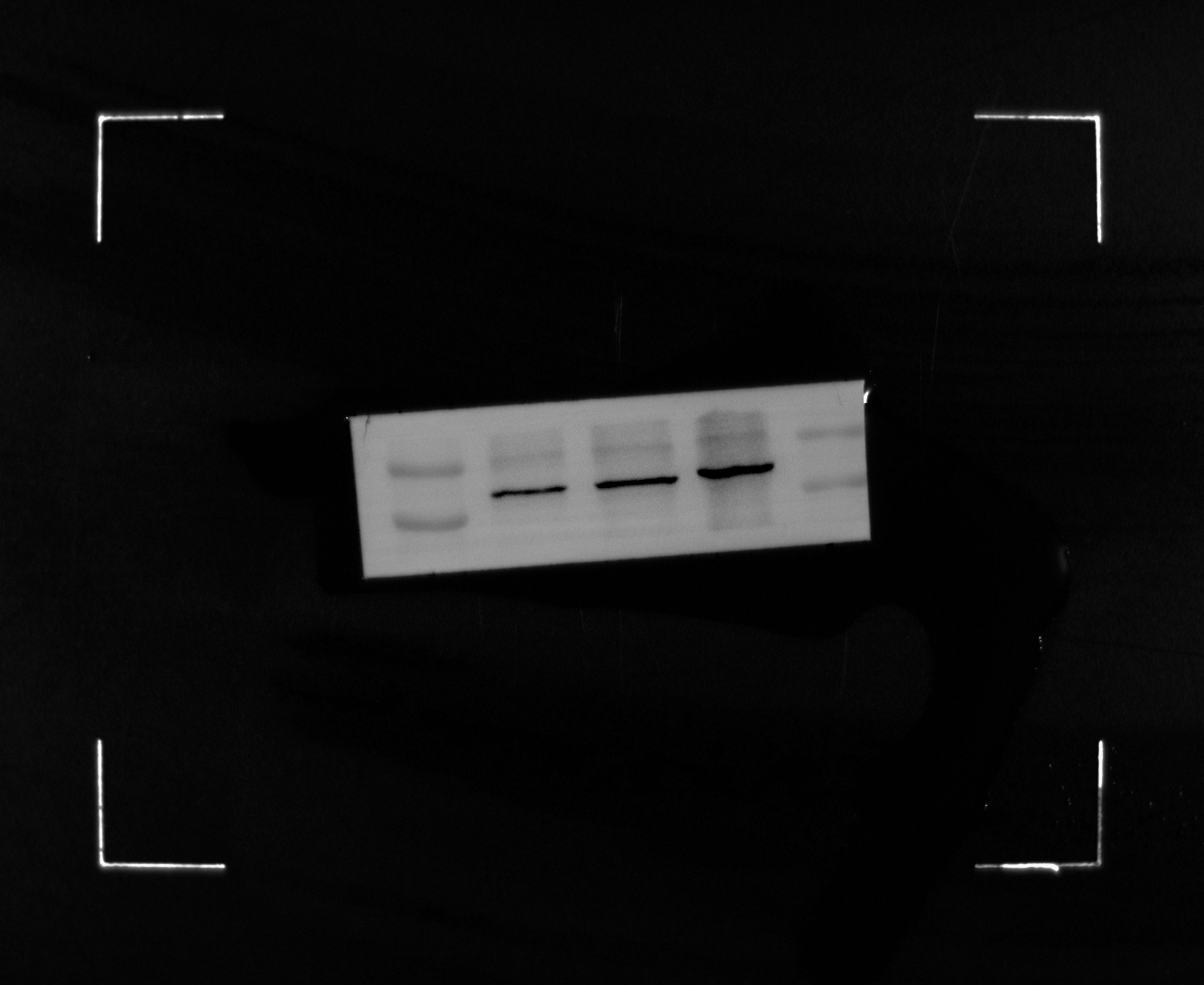

Supplement: Supplementary file 1 [file molecules-28-03842-s001.zip › WB original picture/AFP ALB CK18/21d/CK18-6.tif]

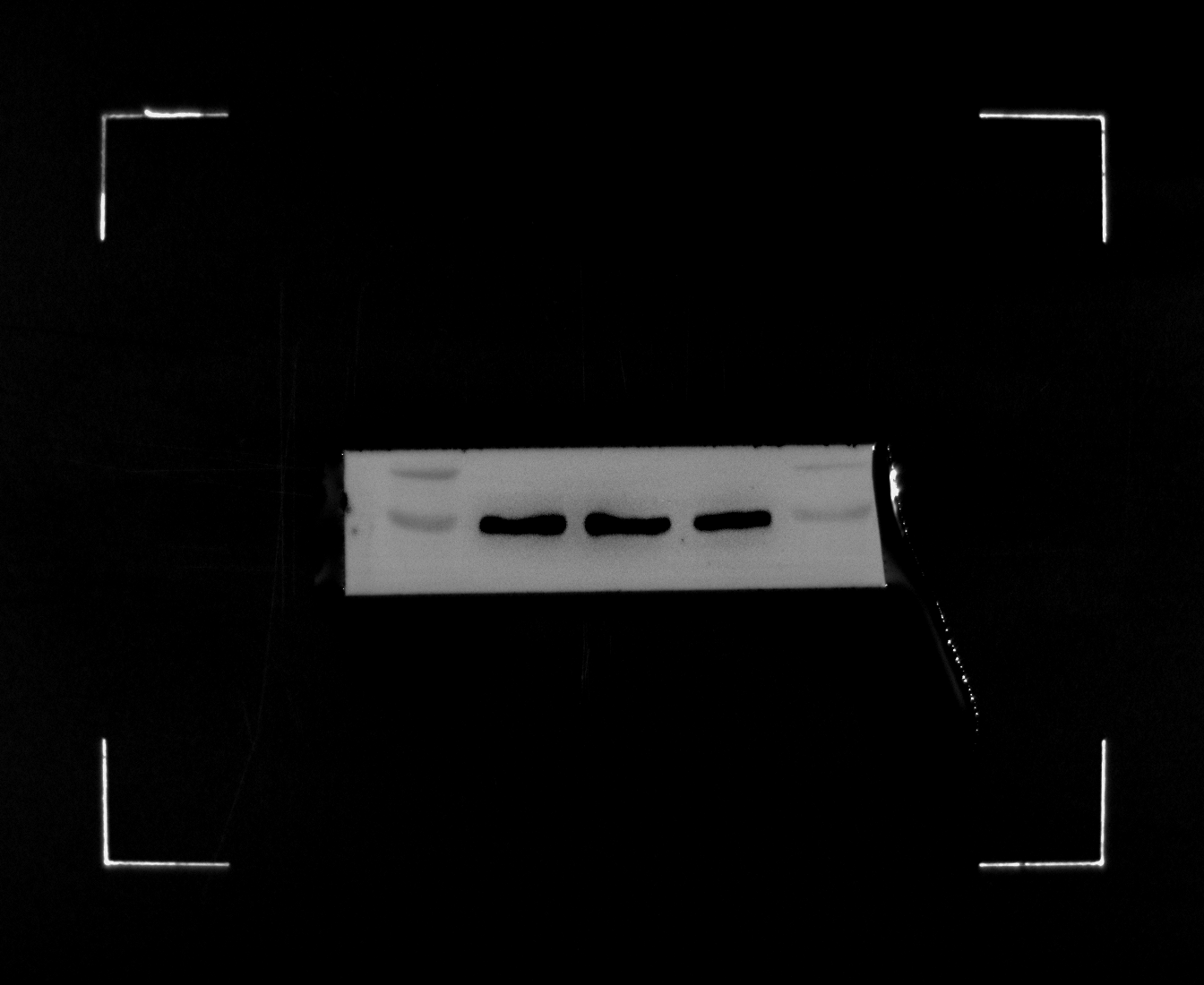

Supplement: Supplementary file 1 [file molecules-28-03842-s001.zip › WB original picture/AFP ALB CK18/21d/GAPDH.tif]

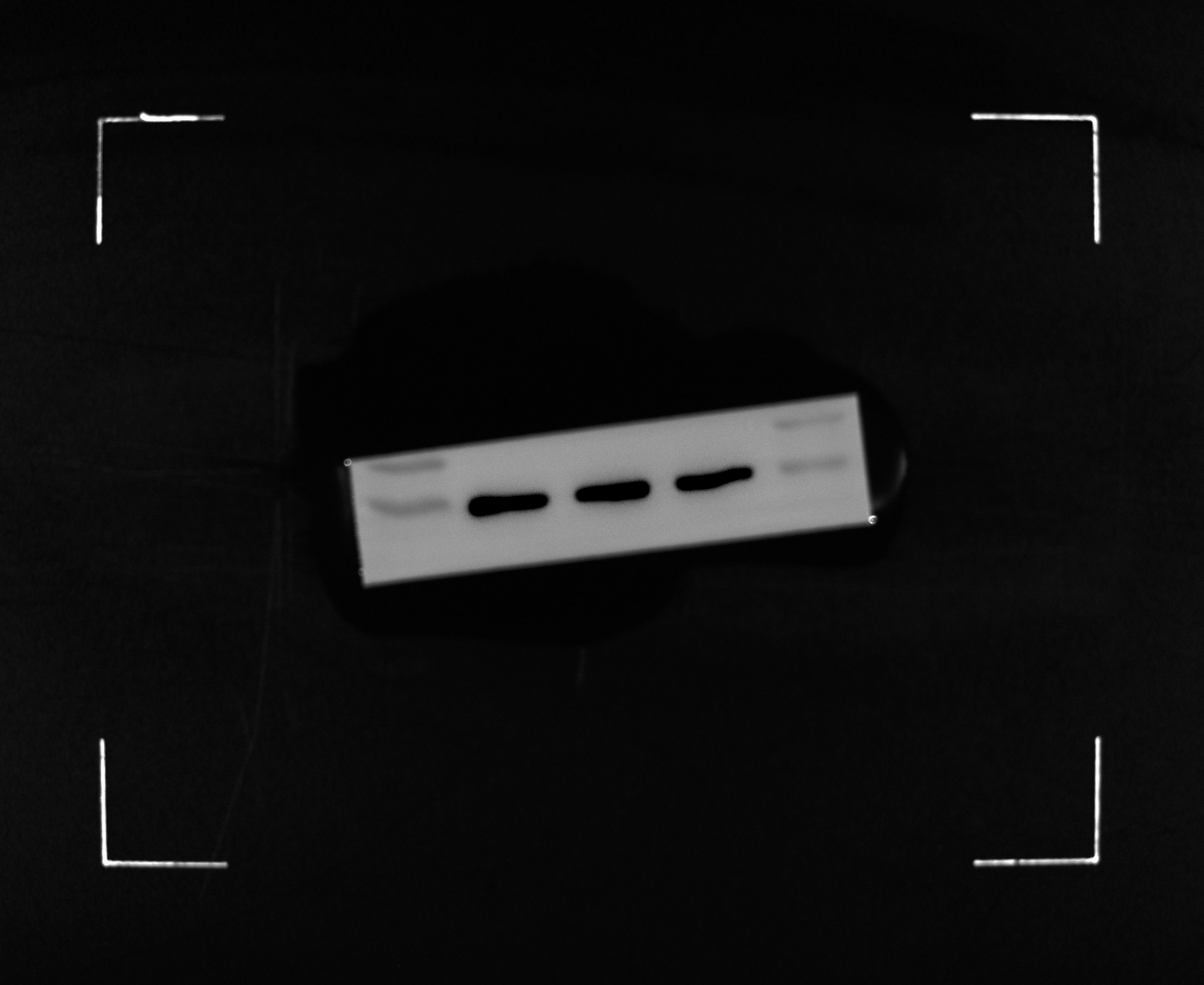

Supplement: Supplementary file 1 [file molecules-28-03842-s001.zip › WB original picture/AFP ALB CK18/21d/GAPDH-2.tif]

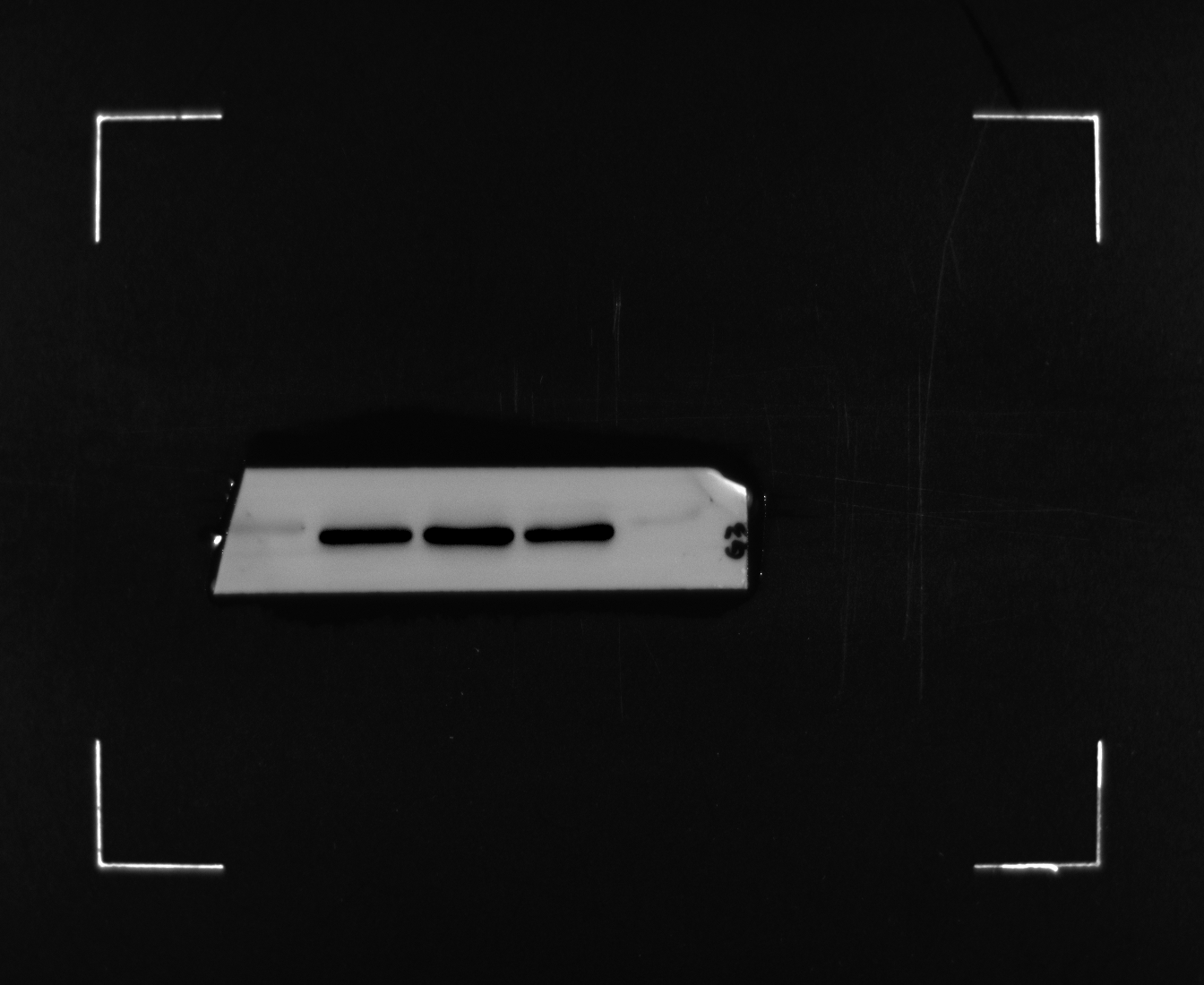

Supplement: Supplementary file 1 [file molecules-28-03842-s001.zip › WB original picture/AFP ALB CK18/21d/GAPDH-3.tif]

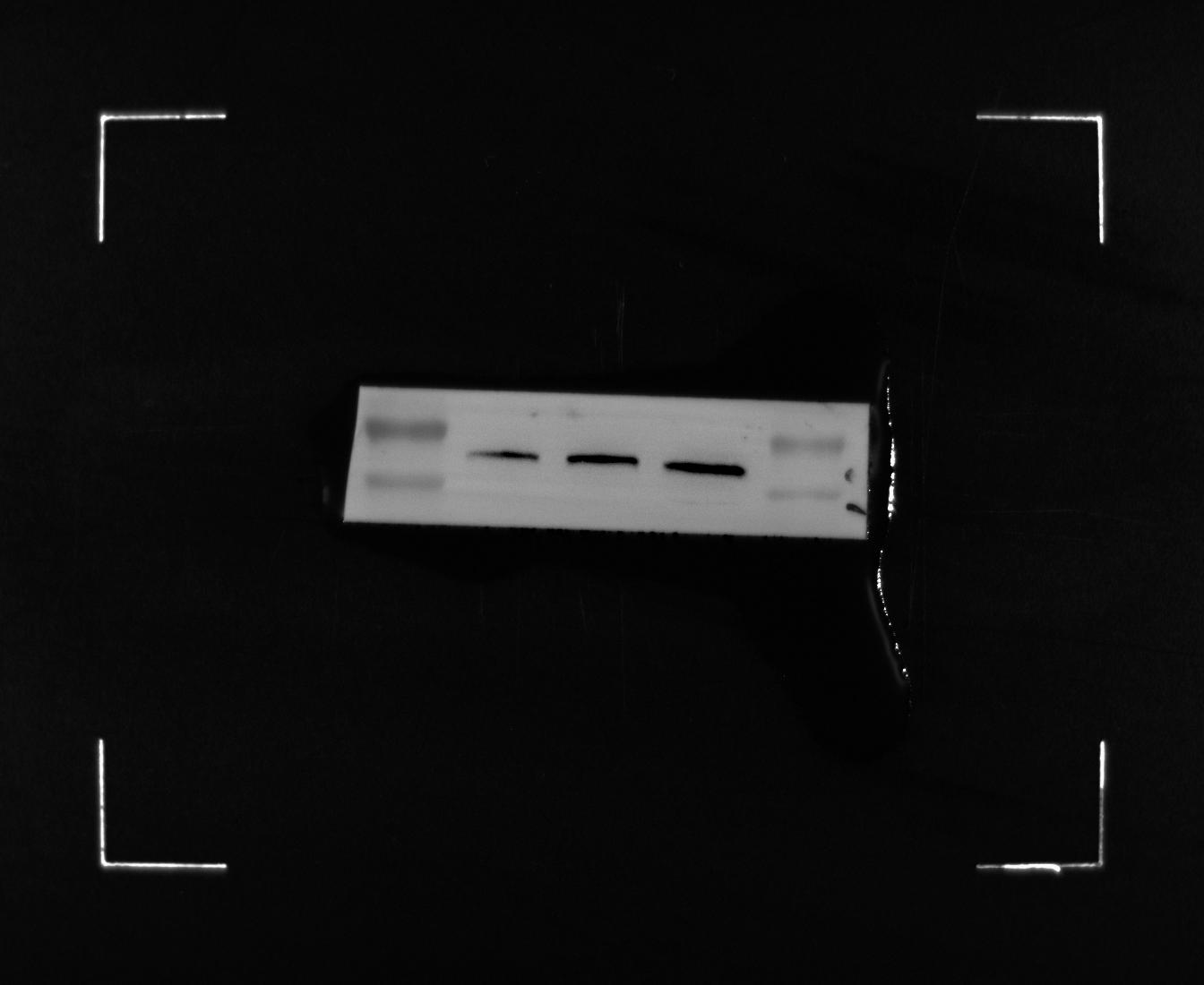

Supplement: Supplementary file 1 [file molecules-28-03842-s001.zip › WB original picture/AFP ALB CK18/7d/AFP-1.tif]

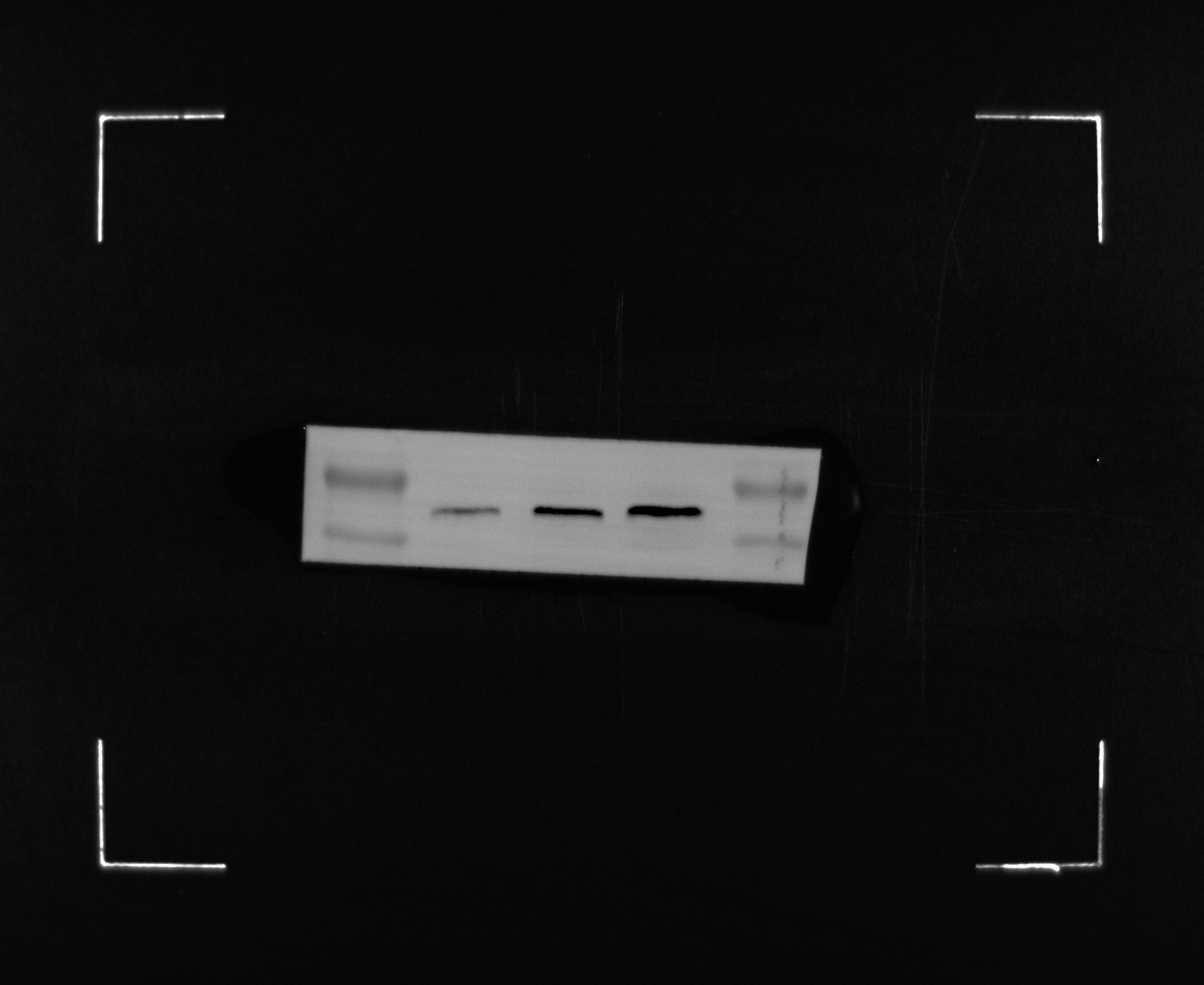

Supplement: Supplementary file 1 [file molecules-28-03842-s001.zip › WB original picture/AFP ALB CK18/7d/AFP-2.tif]

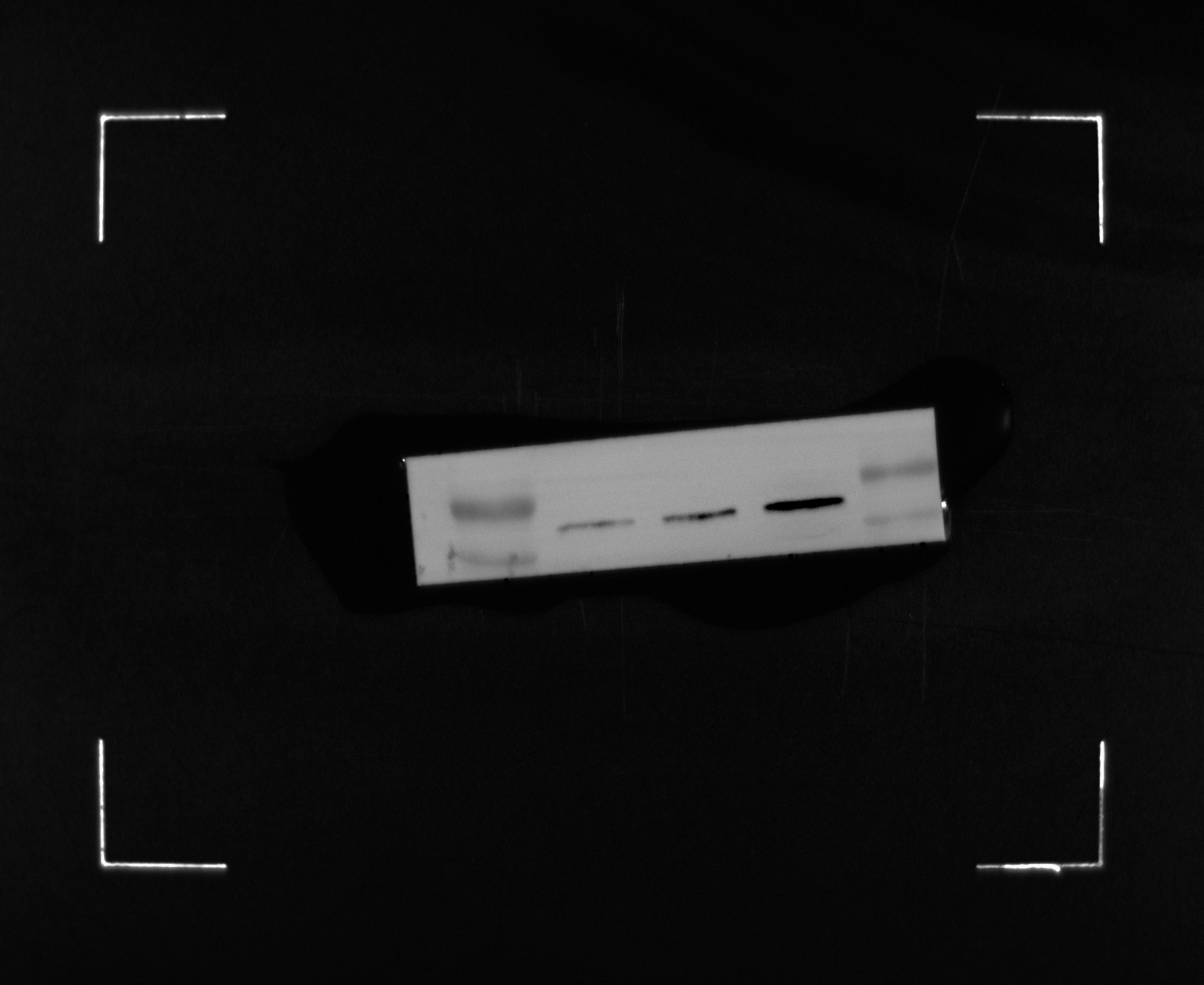

Supplement: Supplementary file 1 [file molecules-28-03842-s001.zip › WB original picture/AFP ALB CK18/7d/AFP-4.tif]

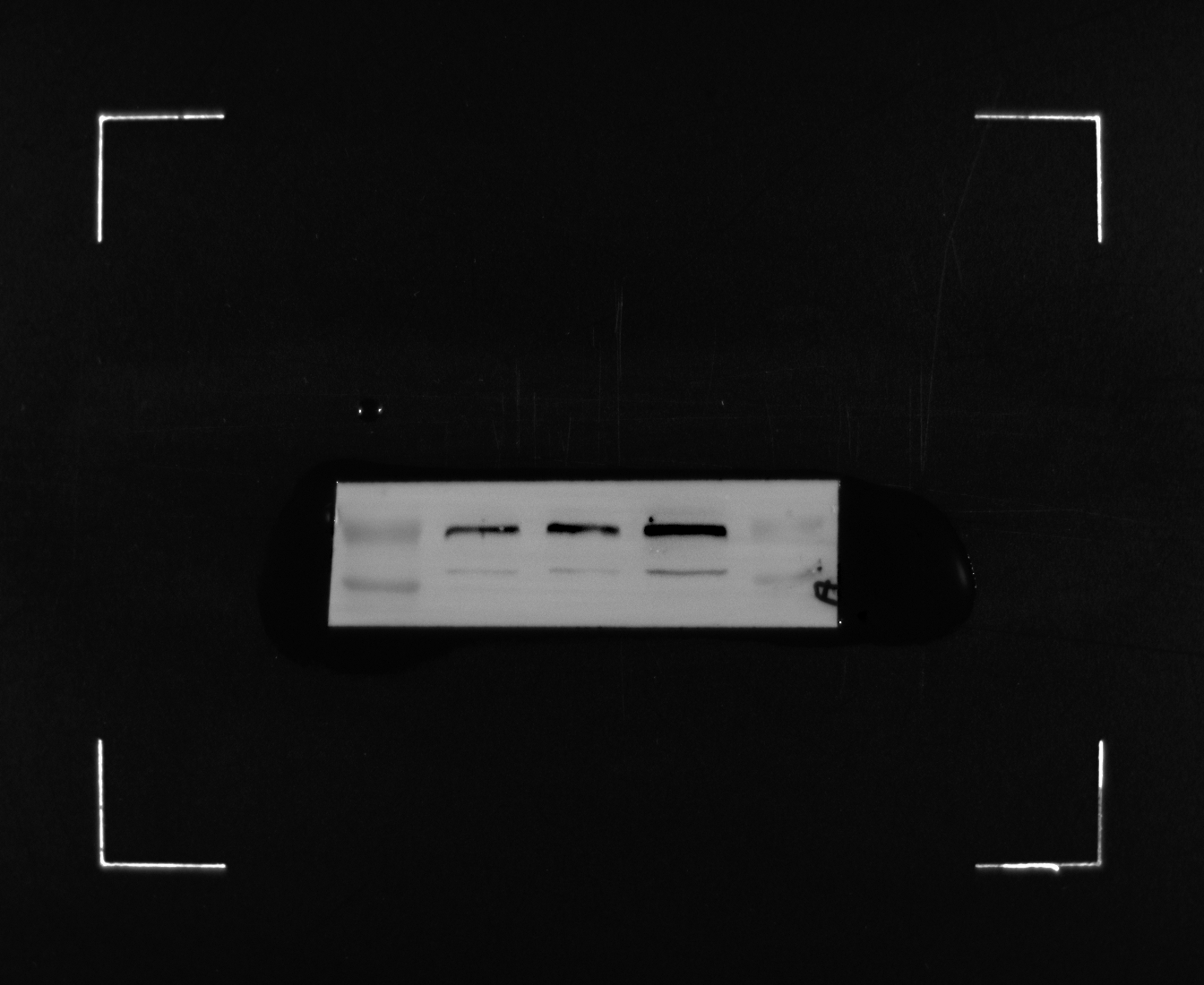

Supplement: Supplementary file 1 [file molecules-28-03842-s001.zip › WB original picture/AFP ALB CK18/7d/ALB.tif]

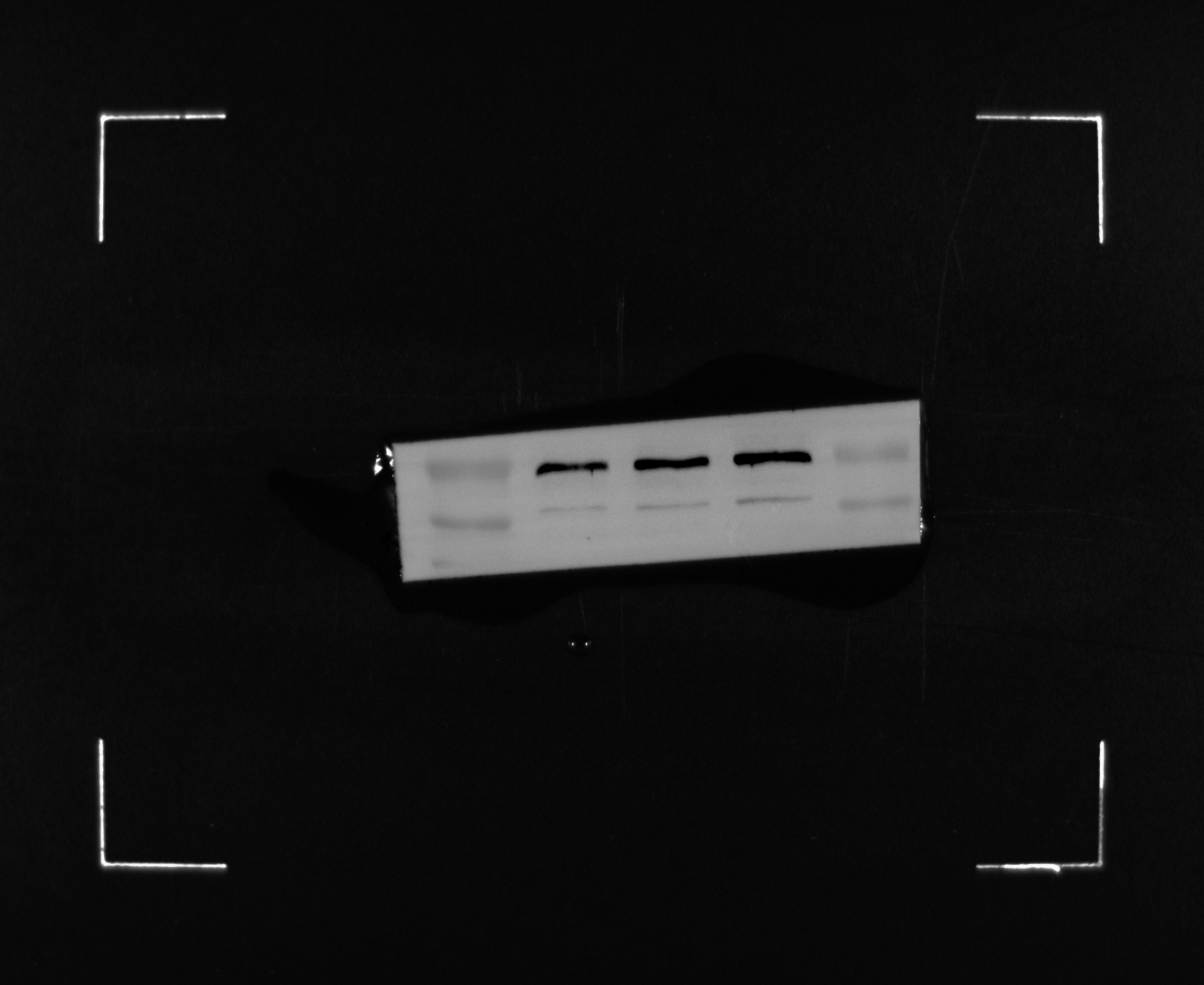

Supplement: Supplementary file 1 [file molecules-28-03842-s001.zip › WB original picture/AFP ALB CK18/7d/ALB-1.tif]

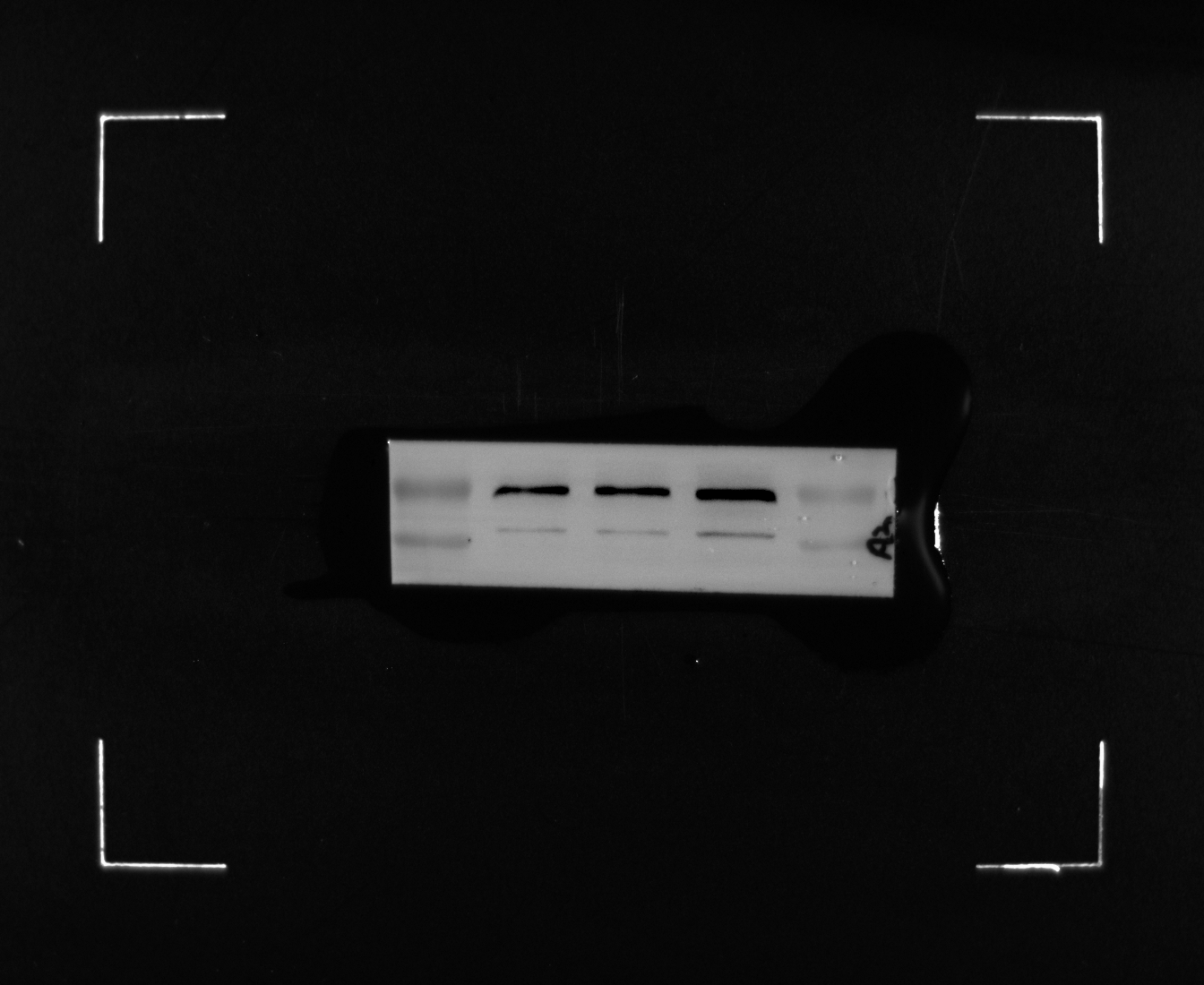

Supplement: Supplementary file 1 [file molecules-28-03842-s001.zip › WB original picture/AFP ALB CK18/7d/ALB-2.tif]

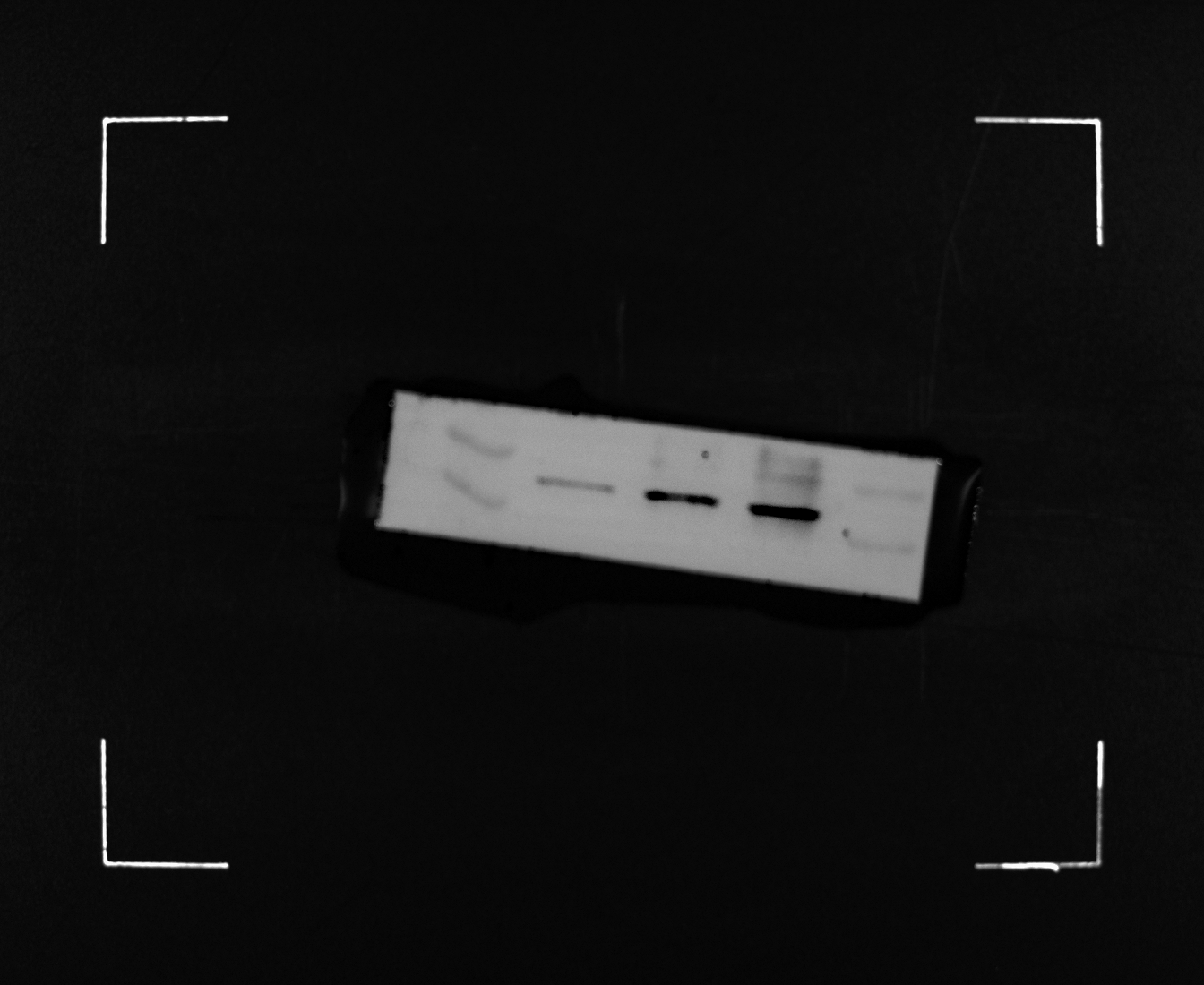

Supplement: Supplementary file 1 [file molecules-28-03842-s001.zip › WB original picture/AFP ALB CK18/7d/CK18.tif]

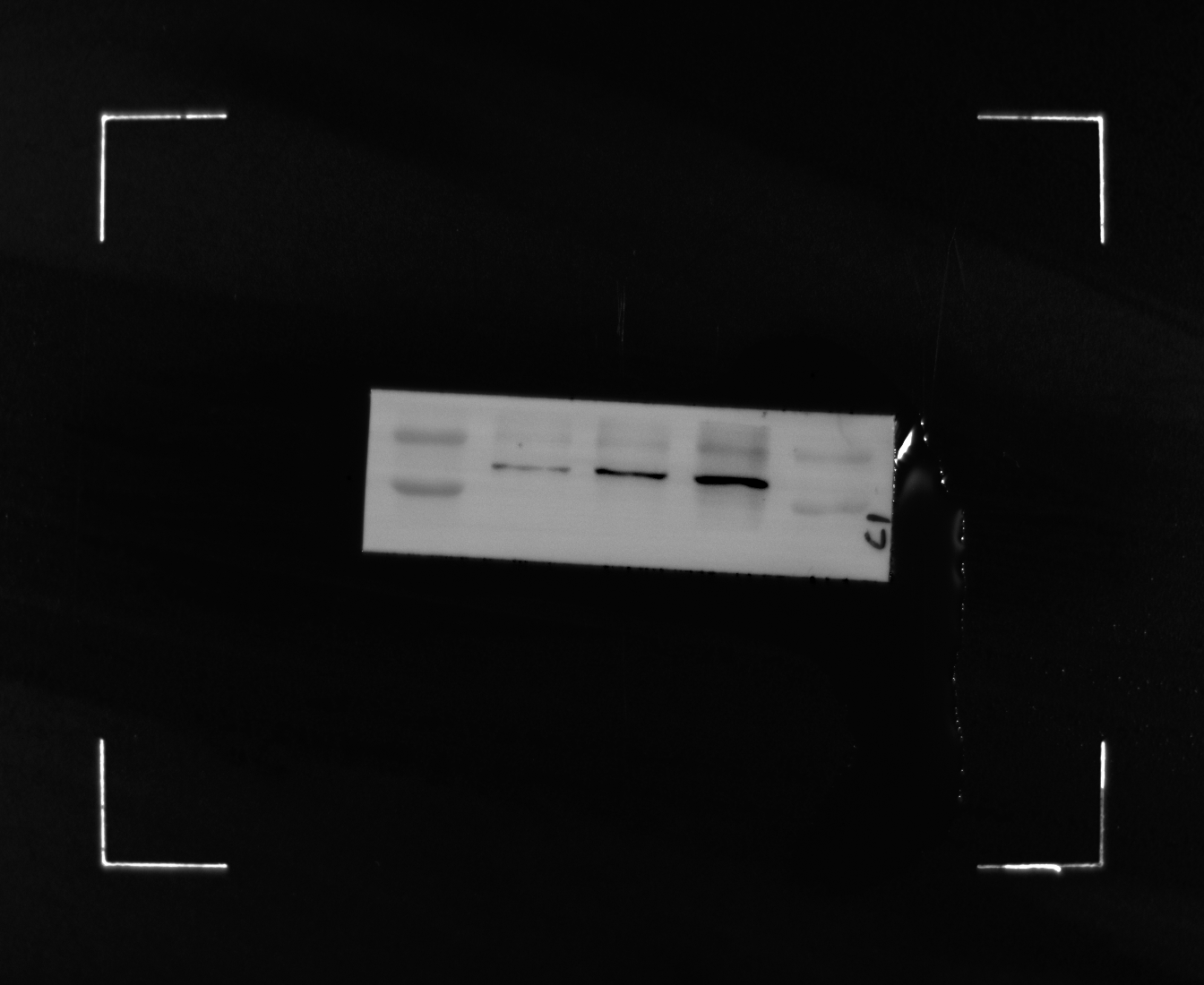

Supplement: Supplementary file 1 [file molecules-28-03842-s001.zip › WB original picture/AFP ALB CK18/7d/CK18-1.tif]

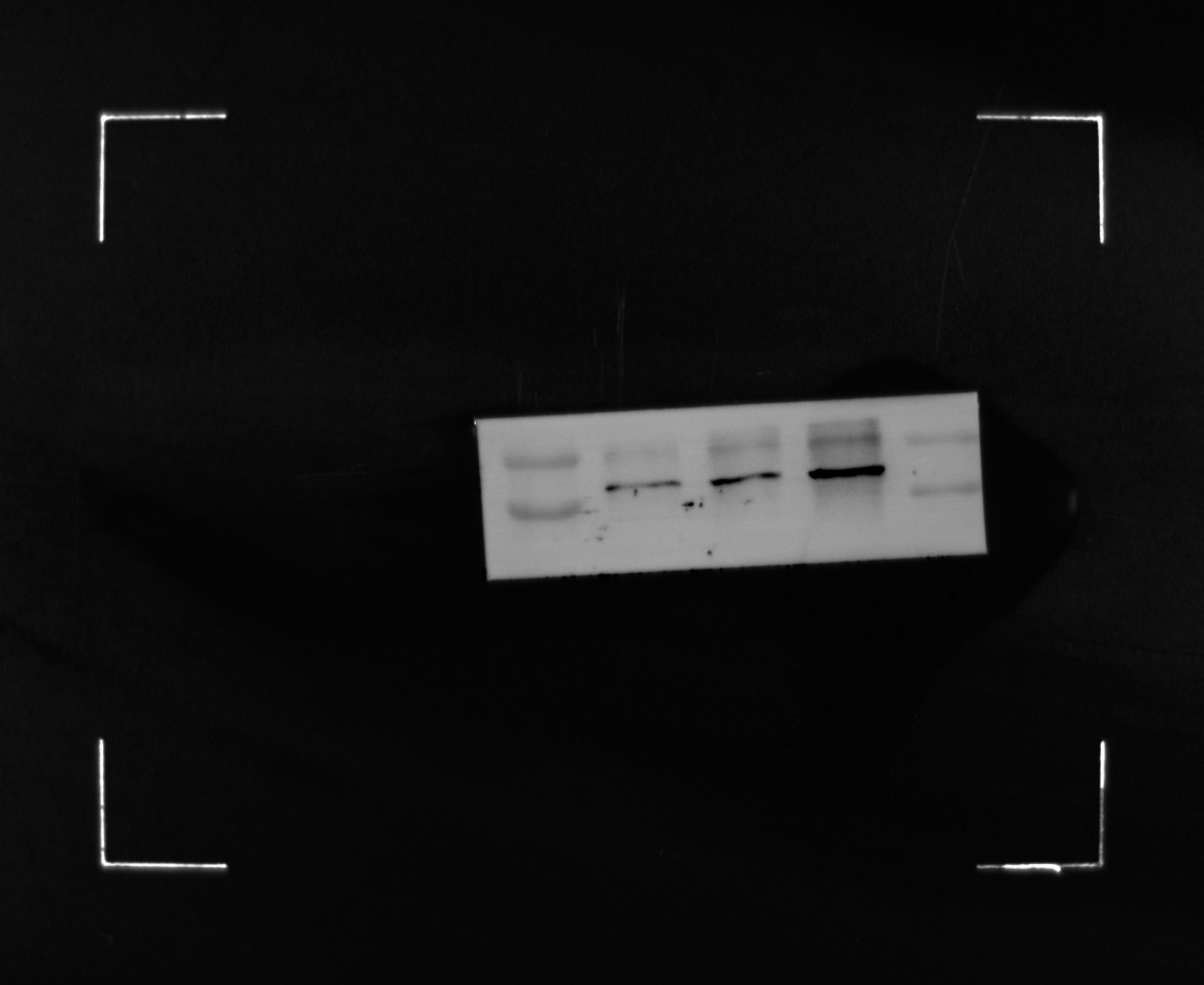

Supplement: Supplementary file 1 [file molecules-28-03842-s001.zip › WB original picture/AFP ALB CK18/7d/CK18-2.tif]

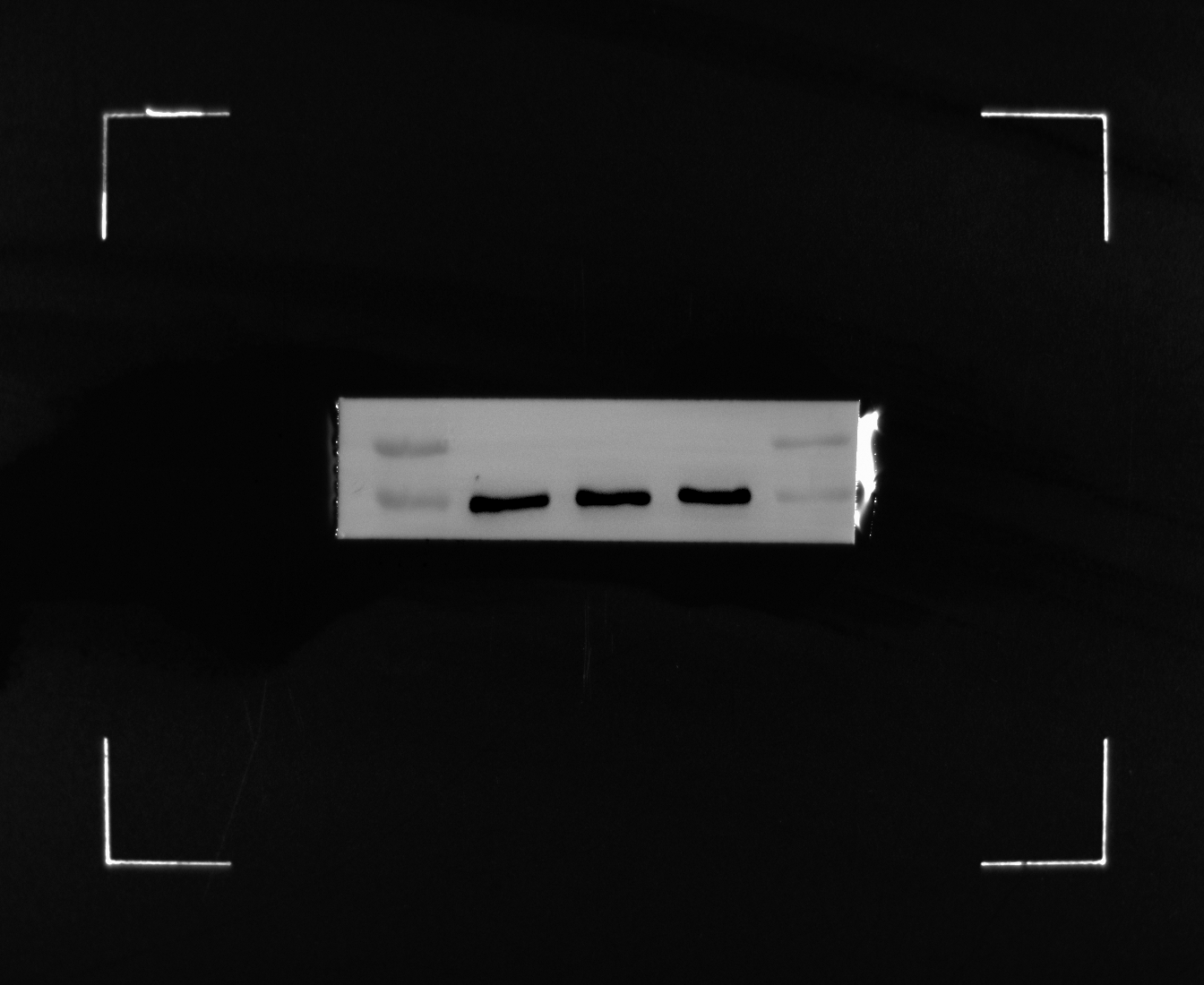

Supplement: Supplementary file 1 [file molecules-28-03842-s001.zip › WB original picture/AFP ALB CK18/7d/GAPDH.tif]

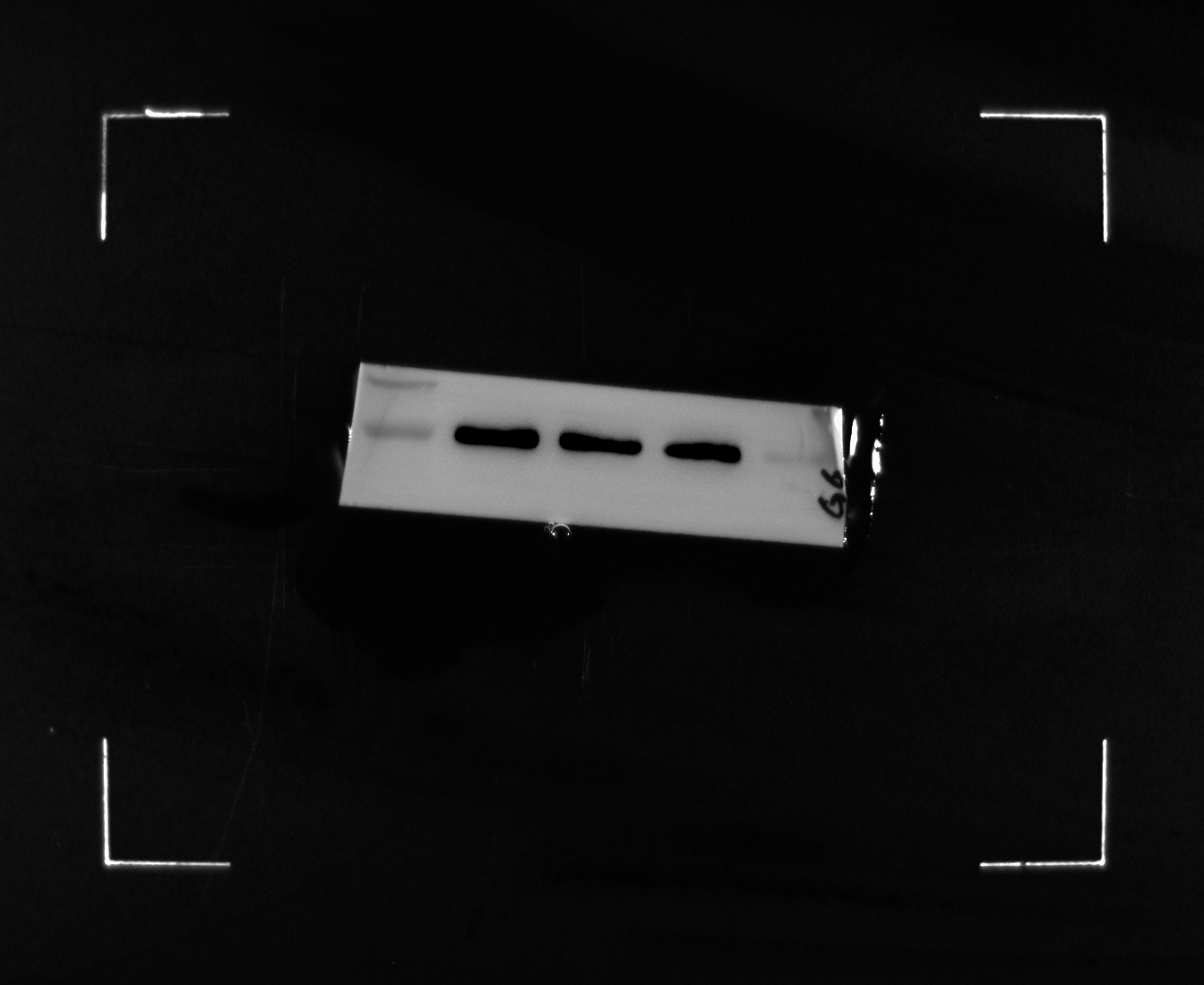

Supplement: Supplementary file 1 [file molecules-28-03842-s001.zip › WB original picture/AFP ALB CK18/7d/GAPDH-6.tif]

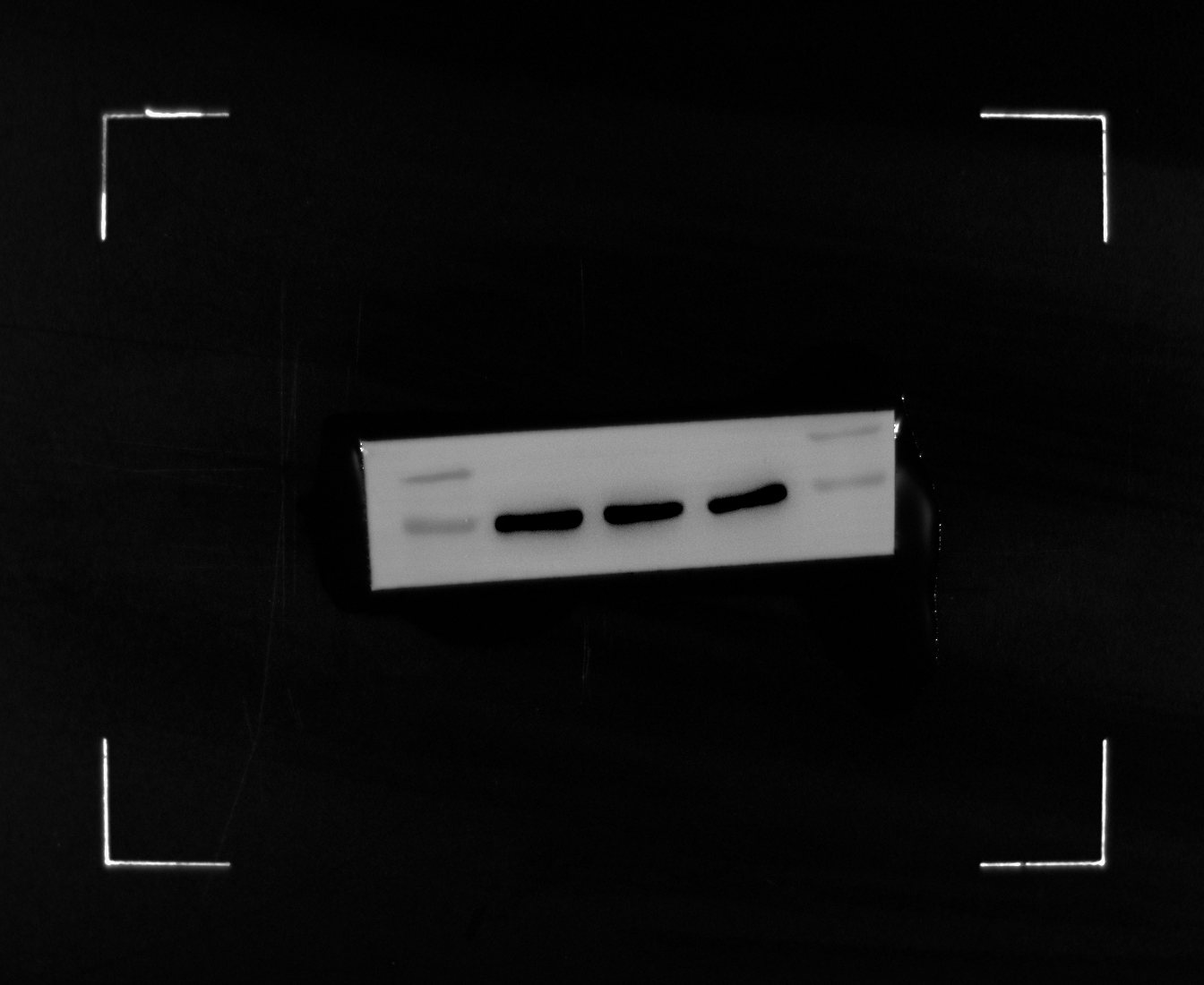

Supplement: Supplementary file 1 [file molecules-28-03842-s001.zip › WB original picture/AFP ALB CK18/7d/GAPDH-7.tif]

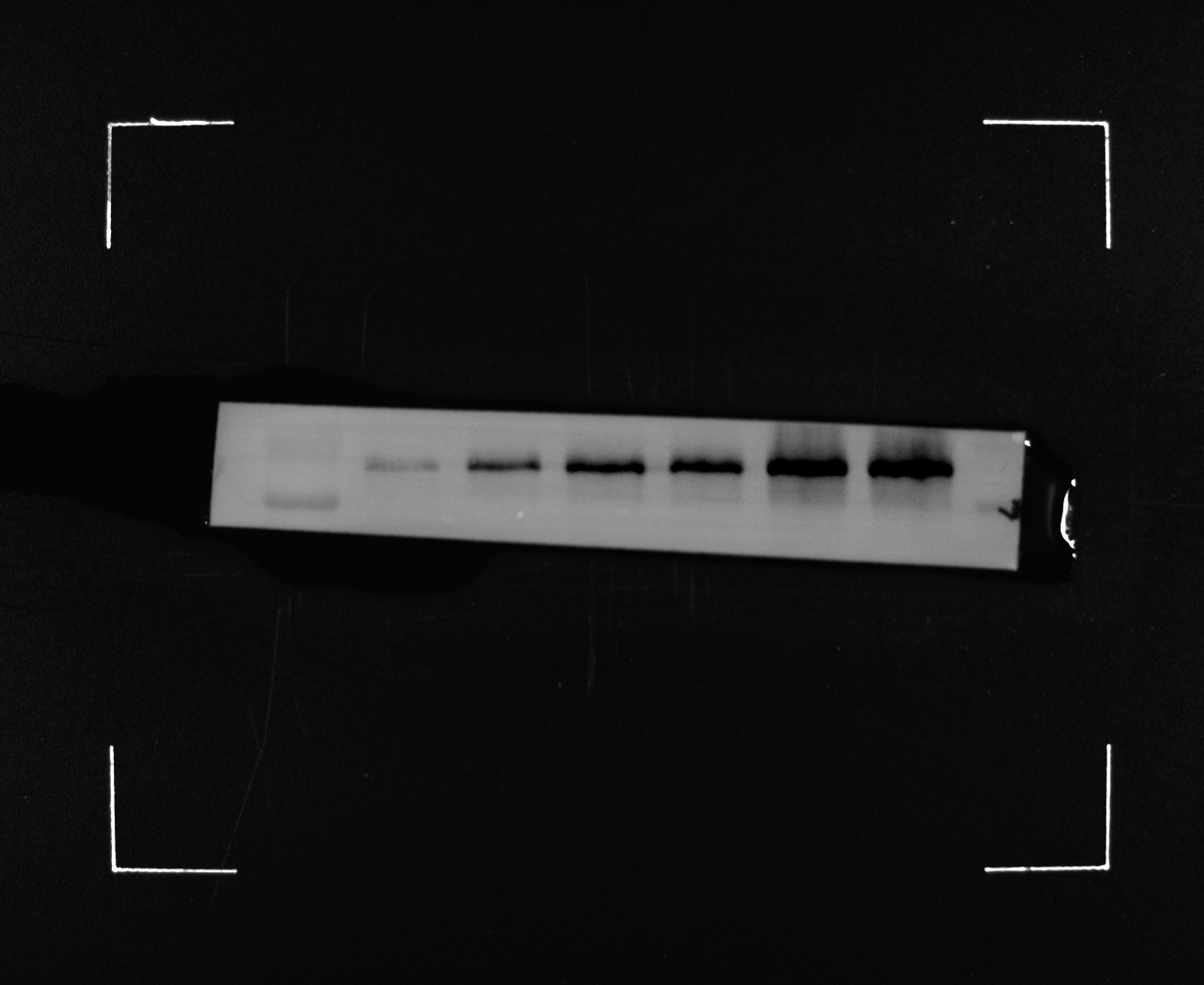

Supplement: Supplementary file 1 [file molecules-28-03842-s001.zip › WB original picture/SDF-1 CXCR4 a┬-catenin p-GSK3a┬/CXCR4_220729_130904_00.01.000_0_10235.tif]

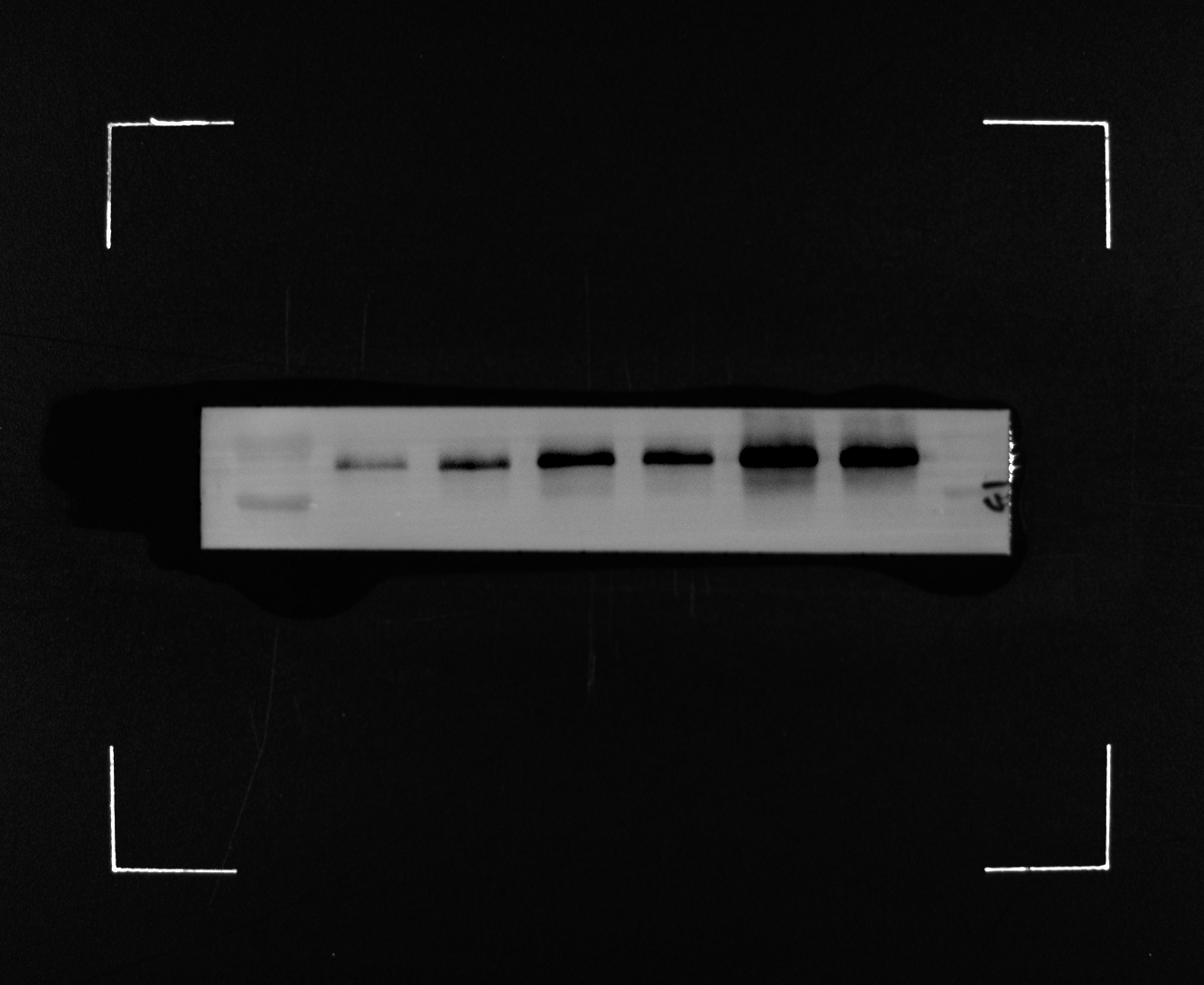

Supplement: Supplementary file 1 [file molecules-28-03842-s001.zip › WB original picture/SDF-1 CXCR4 a┬-catenin p-GSK3a┬/CXCR4-1_220729_130959_00.01.000_0_7665.tif]

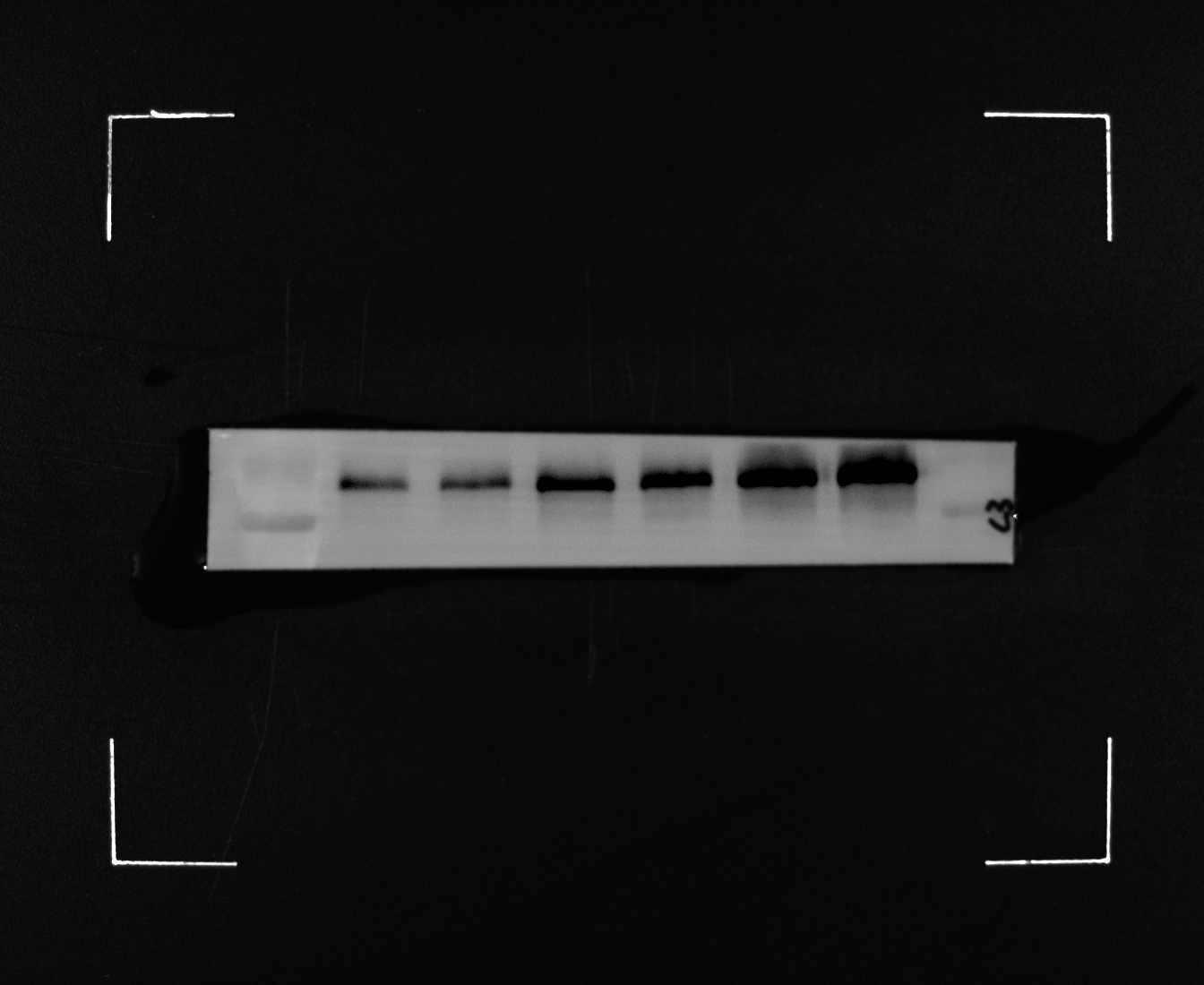

Supplement: Supplementary file 1 [file molecules-28-03842-s001.zip › WB original picture/SDF-1 CXCR4 a┬-catenin p-GSK3a┬/CXCR4-3_220729_131126_00.01.000_0_9509.tif]

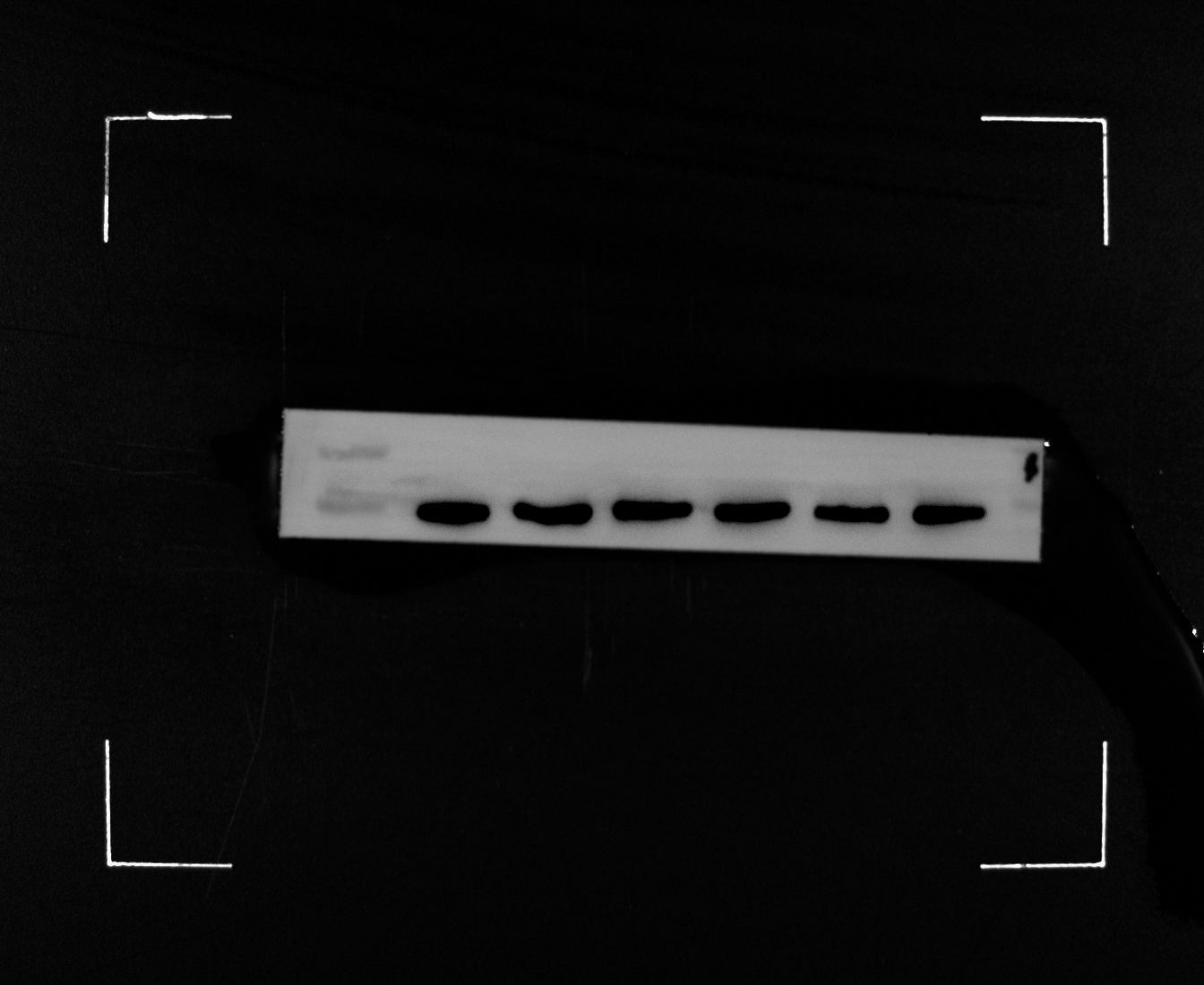

Supplement: Supplementary file 1 [file molecules-28-03842-s001.zip › WB original picture/SDF-1 CXCR4 a┬-catenin p-GSK3a┬/GAPDH-1_220727_122455_00.01.000_0_5041.tif]

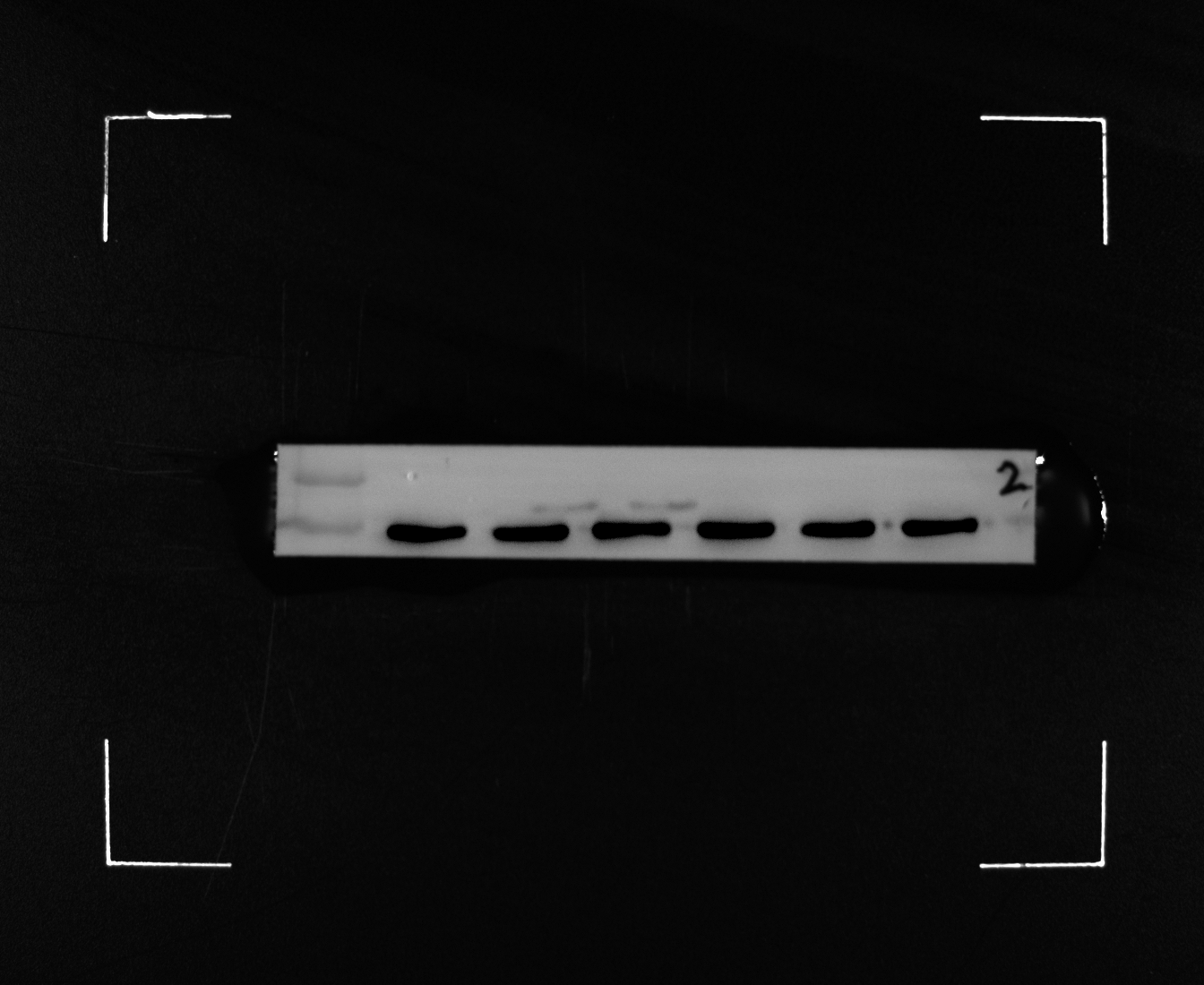

Supplement: Supplementary file 1 [file molecules-28-03842-s001.zip › WB original picture/SDF-1 CXCR4 a┬-catenin p-GSK3a┬/GAPDH-2_220727_122551_00.01.000_0_9080.tif]

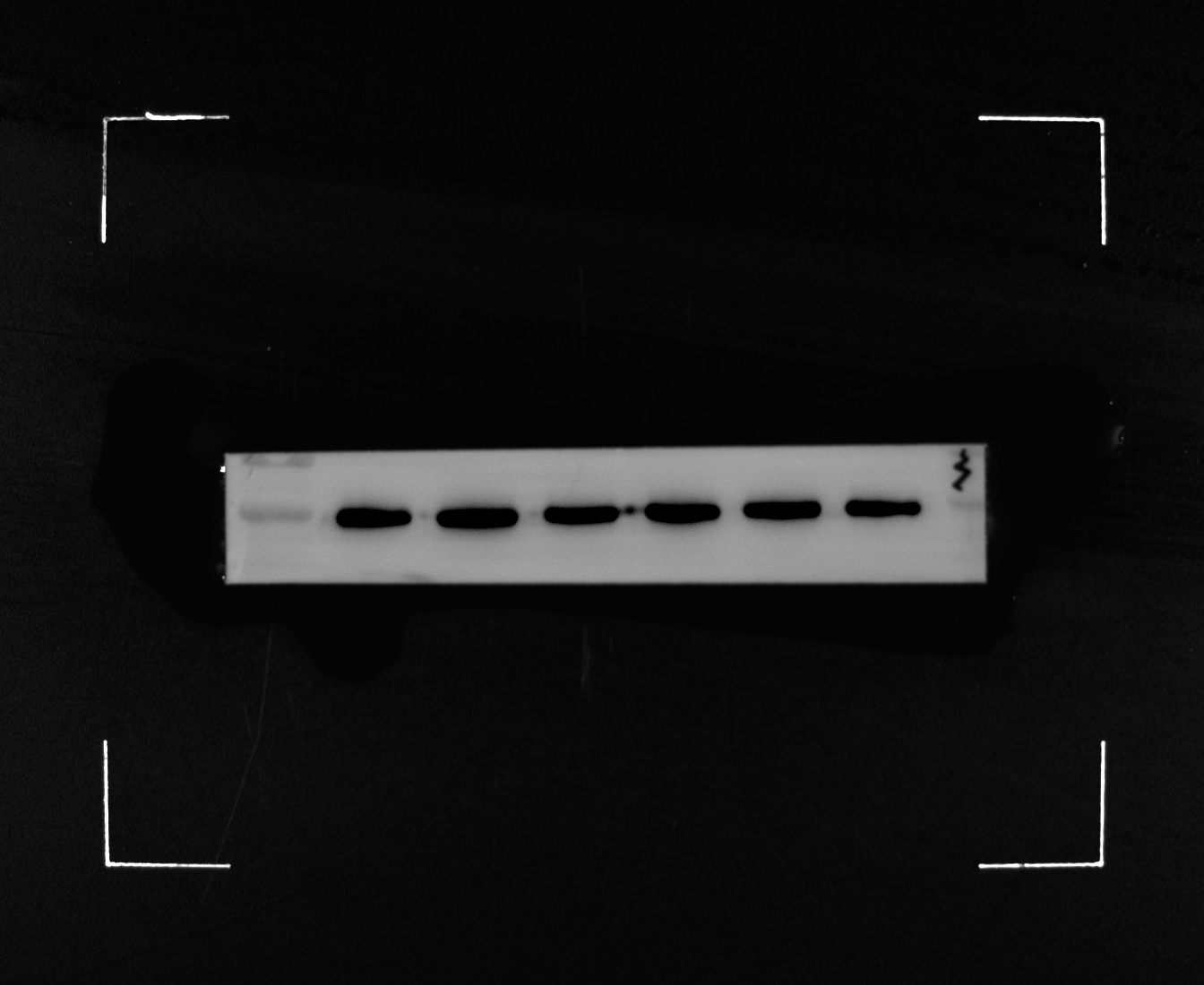

Supplement: Supplementary file 1 [file molecules-28-03842-s001.zip › WB original picture/SDF-1 CXCR4 a┬-catenin p-GSK3a┬/GAPDH-3_220727_122636_00.01.000_0_17791.tif]

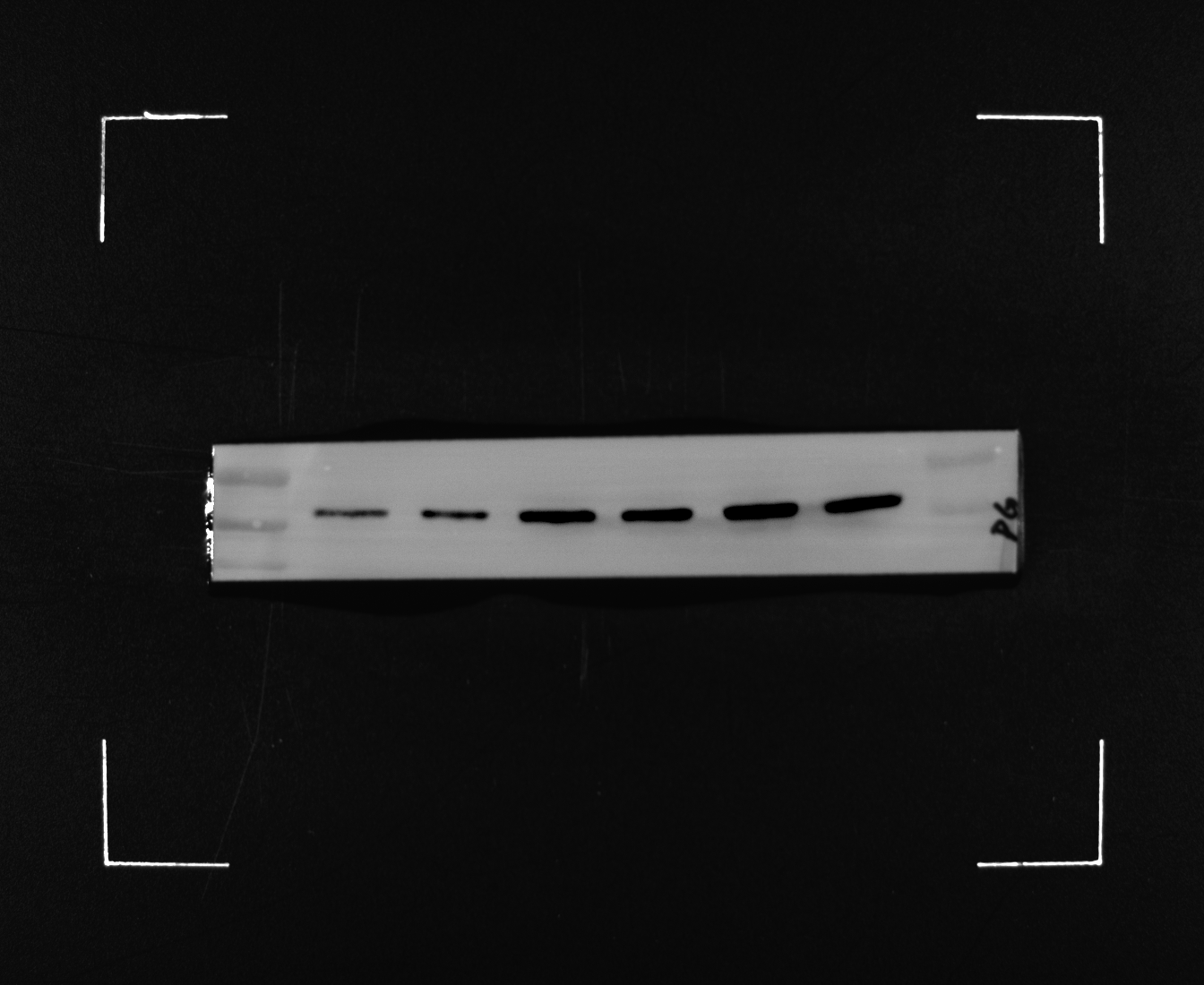

Supplement: Supplementary file 1 [file molecules-28-03842-s001.zip › WB original picture/SDF-1 CXCR4 a┬-catenin p-GSK3a┬/P-GSK3a┬_220729_131225_00.01.000_0_27285.tif]

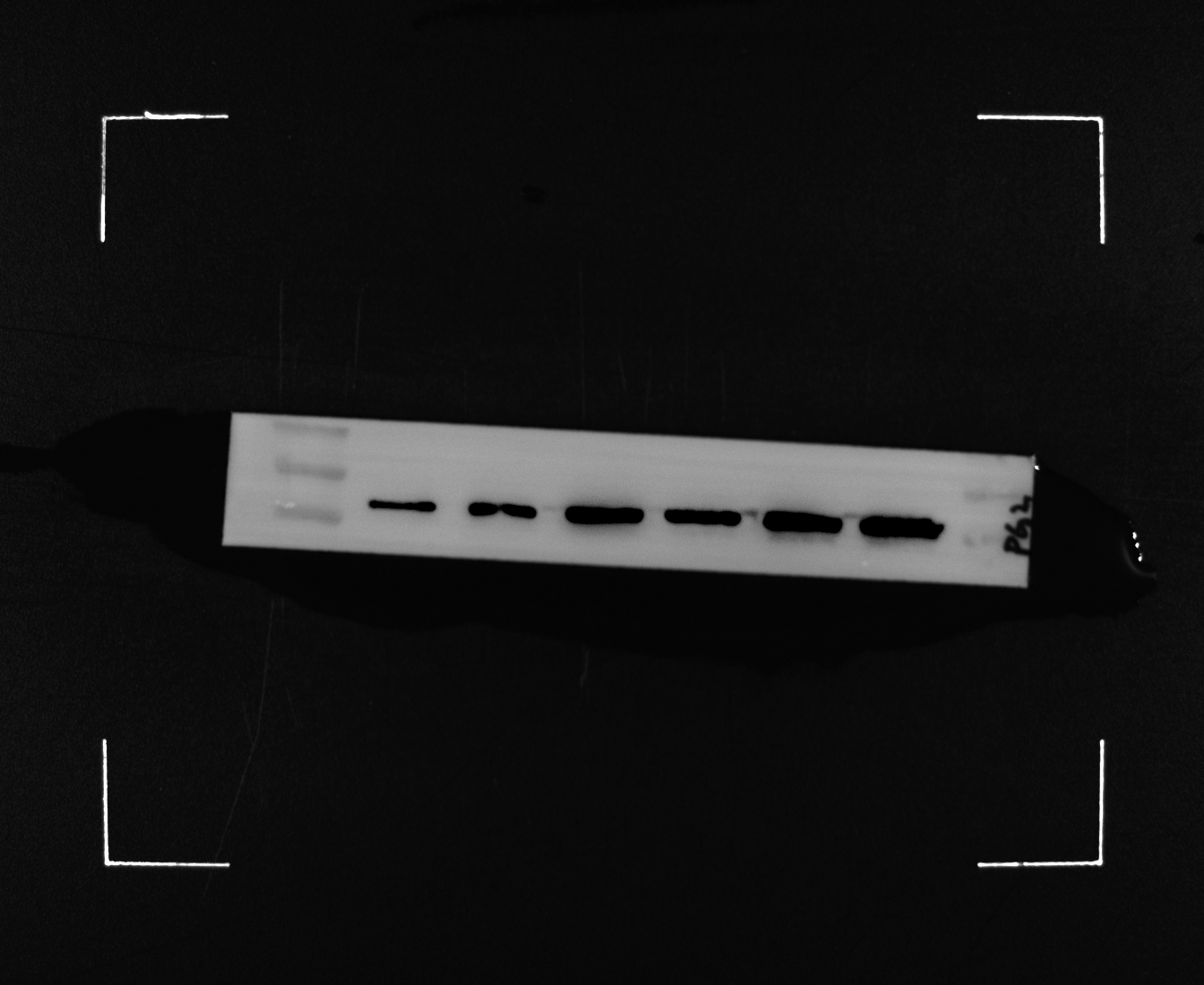

Supplement: Supplementary file 1 [file molecules-28-03842-s001.zip › WB original picture/SDF-1 CXCR4 a┬-catenin p-GSK3a┬/P-GSK3a┬-2_220729_131353_00.01.000_0_12077.tif]

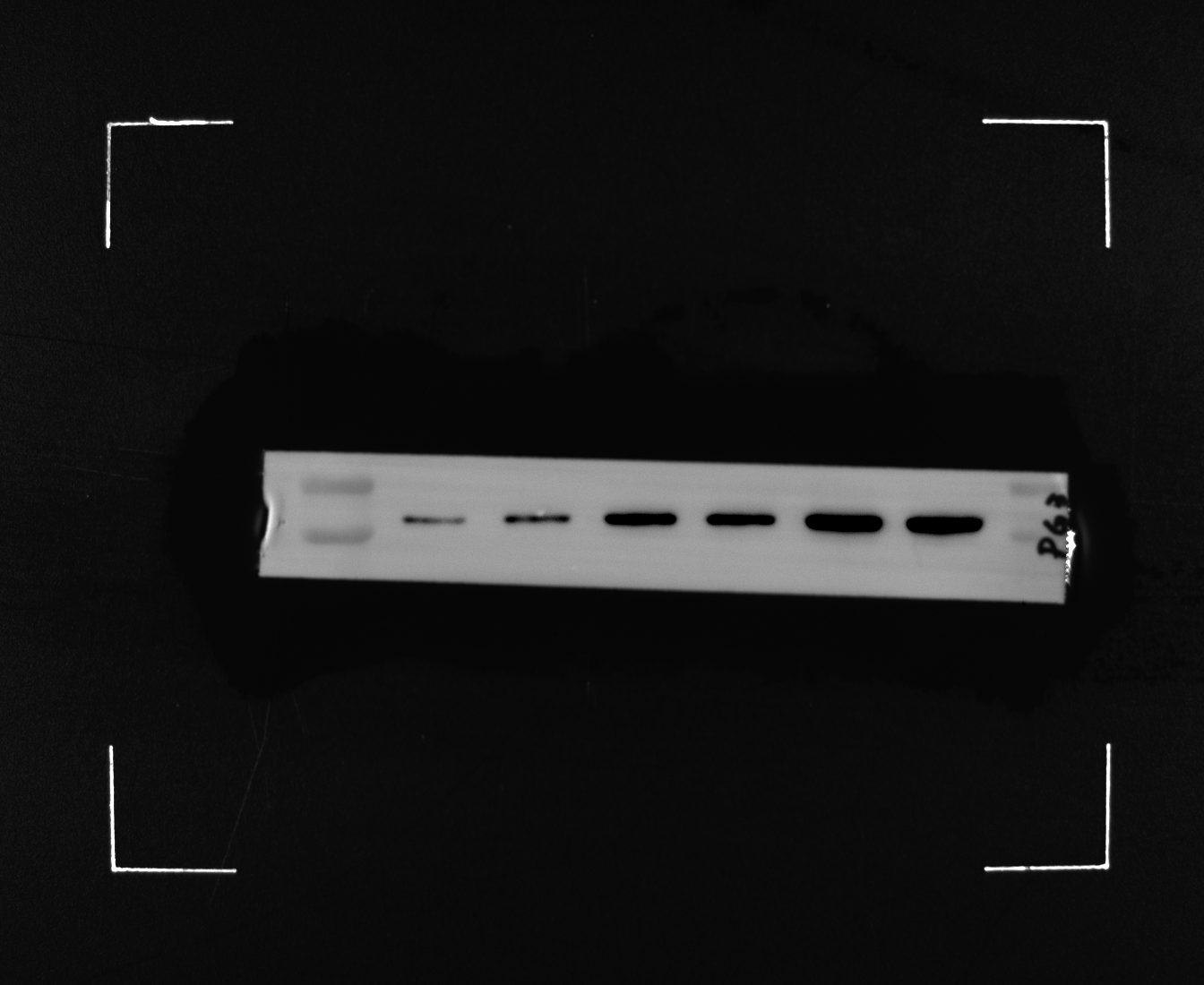

Supplement: Supplementary file 1 [file molecules-28-03842-s001.zip › WB original picture/SDF-1 CXCR4 a┬-catenin p-GSK3a┬/P-GSK3a┬-3_220729_131504_00.01.000_0_20933.tif]

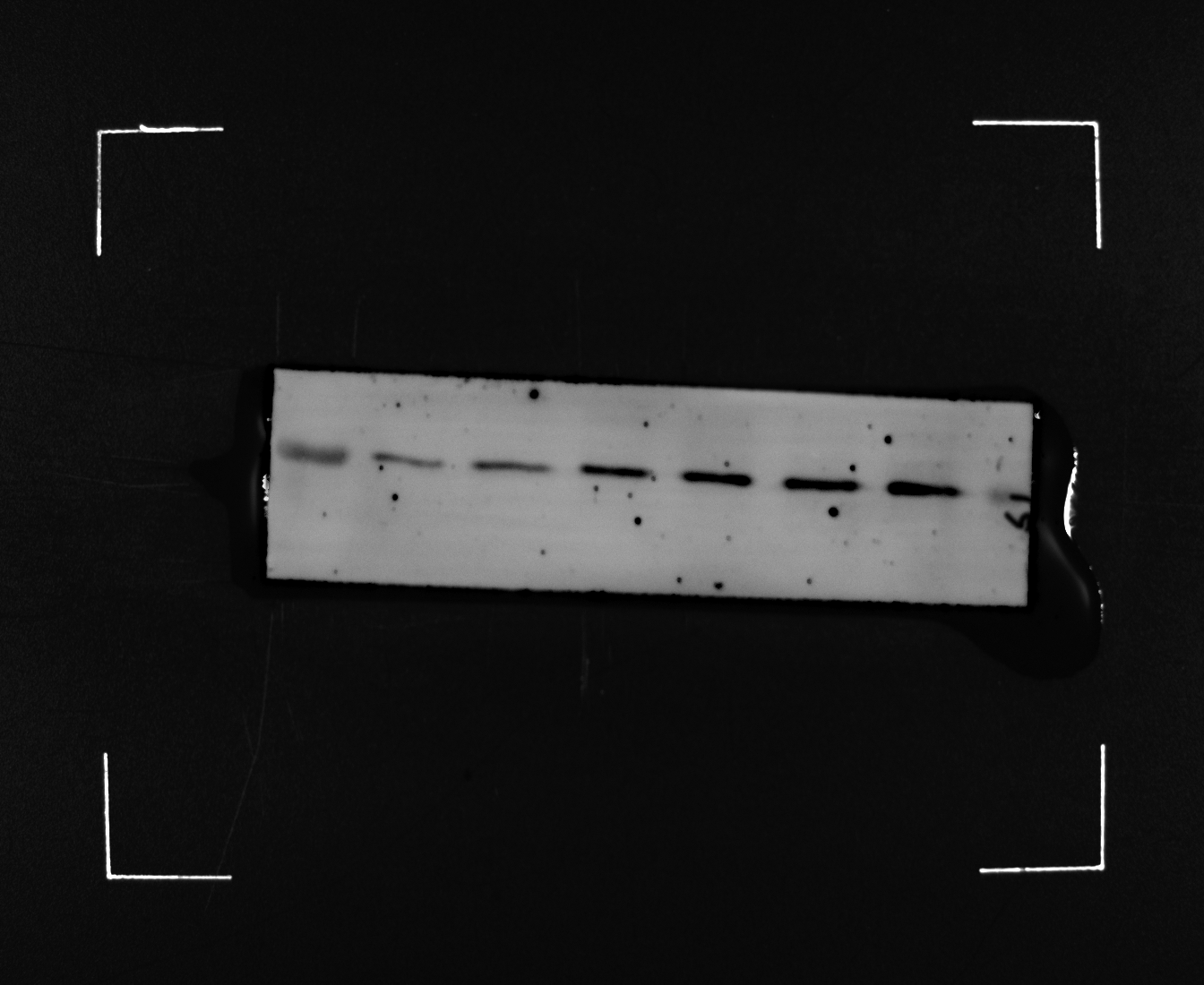

Supplement: Supplementary file 1 [file molecules-28-03842-s001.zip › WB original picture/SDF-1 CXCR4 a┬-catenin p-GSK3a┬/SDF-1-1_220728_111220_00.05.000_0_15352.tif]

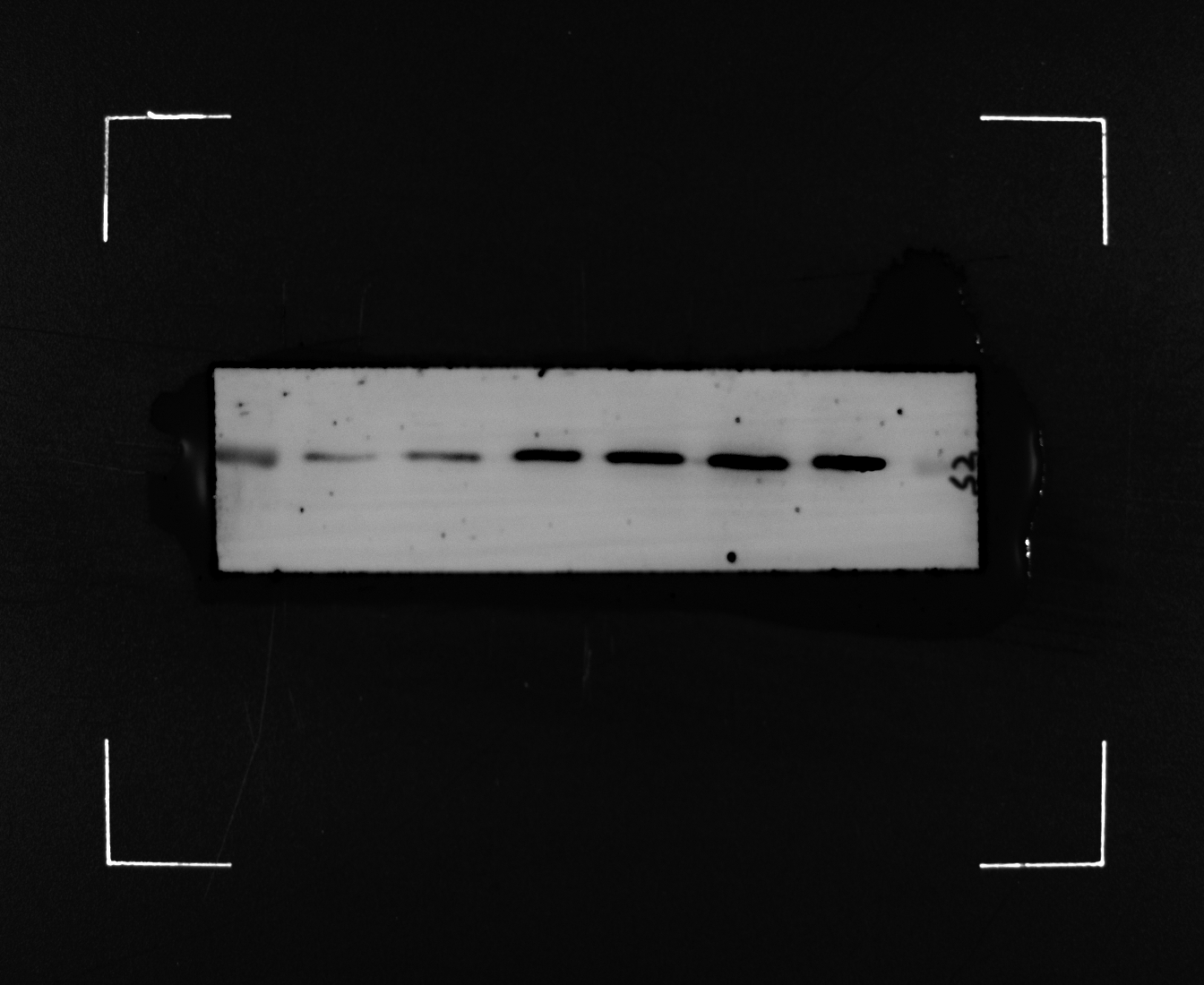

Supplement: Supplementary file 1 [file molecules-28-03842-s001.zip › WB original picture/SDF-1 CXCR4 a┬-catenin p-GSK3a┬/SDF-1-2_220728_111314_00.05.000_0_12019.tif]

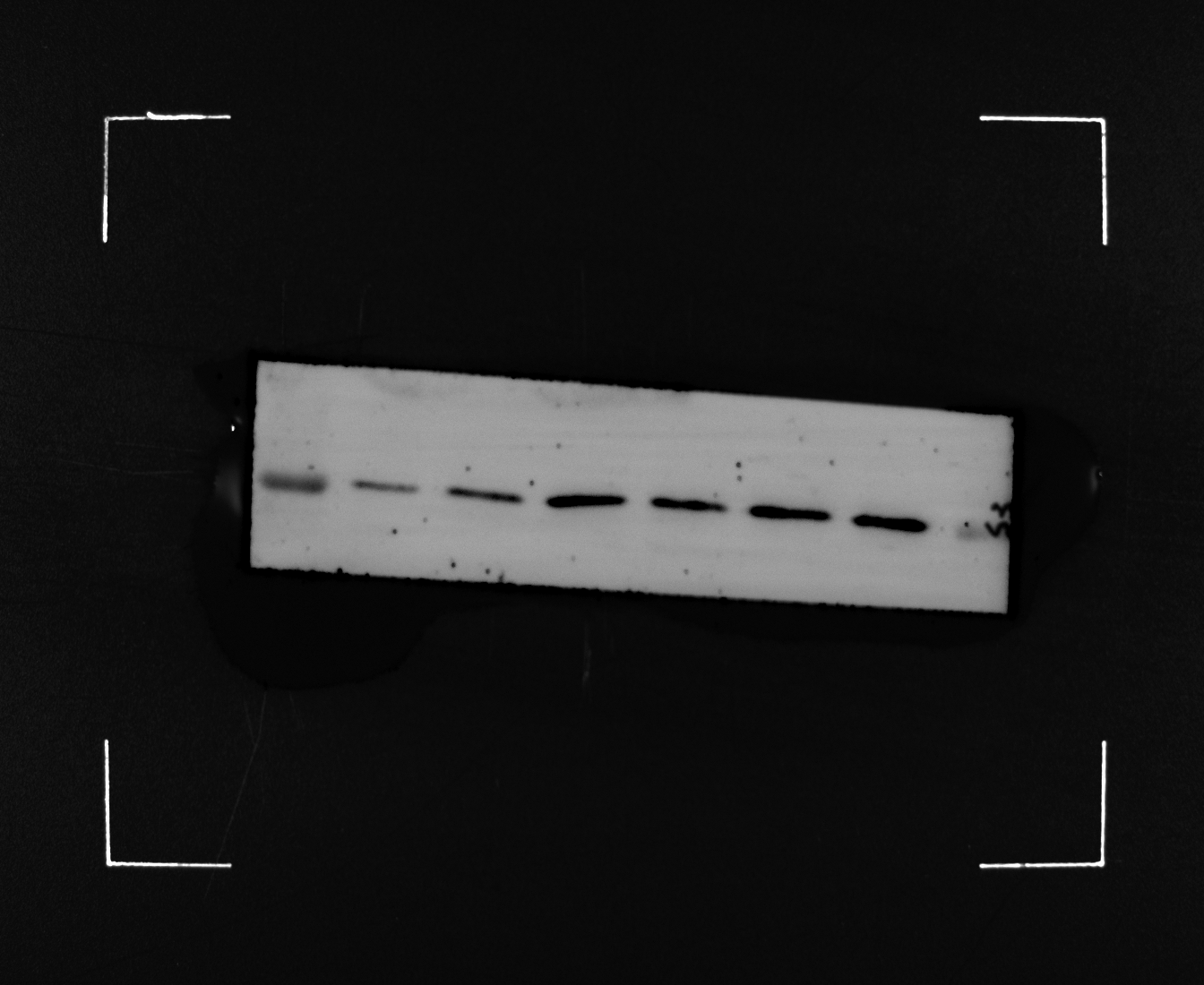

Supplement: Supplementary file 1 [file molecules-28-03842-s001.zip › WB original picture/SDF-1 CXCR4 a┬-catenin p-GSK3a┬/SDF-1-3_220728_111401_00.05.000_0_10011.tif]

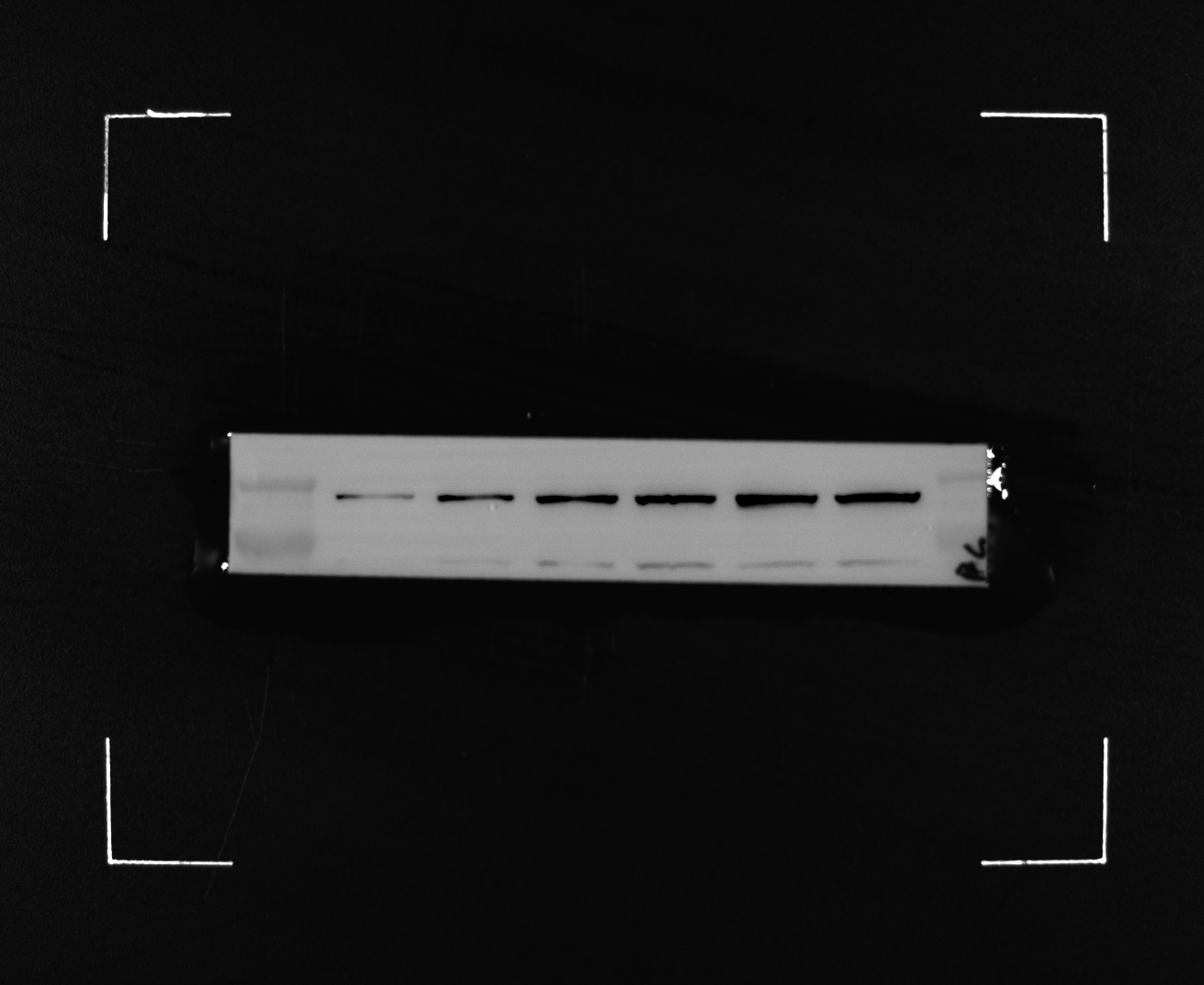

Supplement: Supplementary file 1 [file molecules-28-03842-s001.zip › WB original picture/SDF-1 CXCR4 a┬-catenin p-GSK3a┬/a┬-catenin_220728_111839_00.01.000_0_24848.tif]

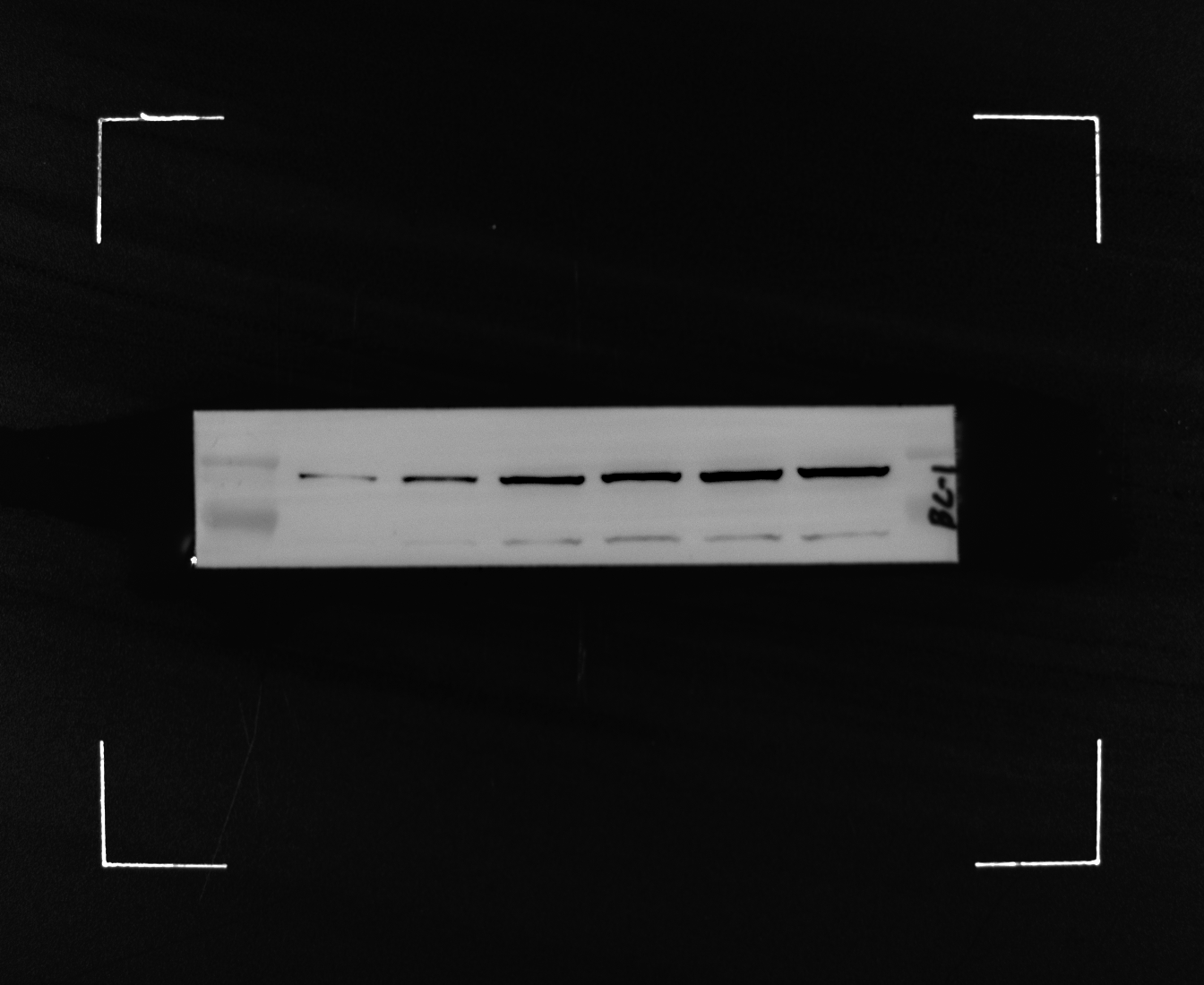

Supplement: Supplementary file 1 [file molecules-28-03842-s001.zip › WB original picture/SDF-1 CXCR4 a┬-catenin p-GSK3a┬/a┬-catenin-1_220728_111915_00.01.000_0_27770.tif]

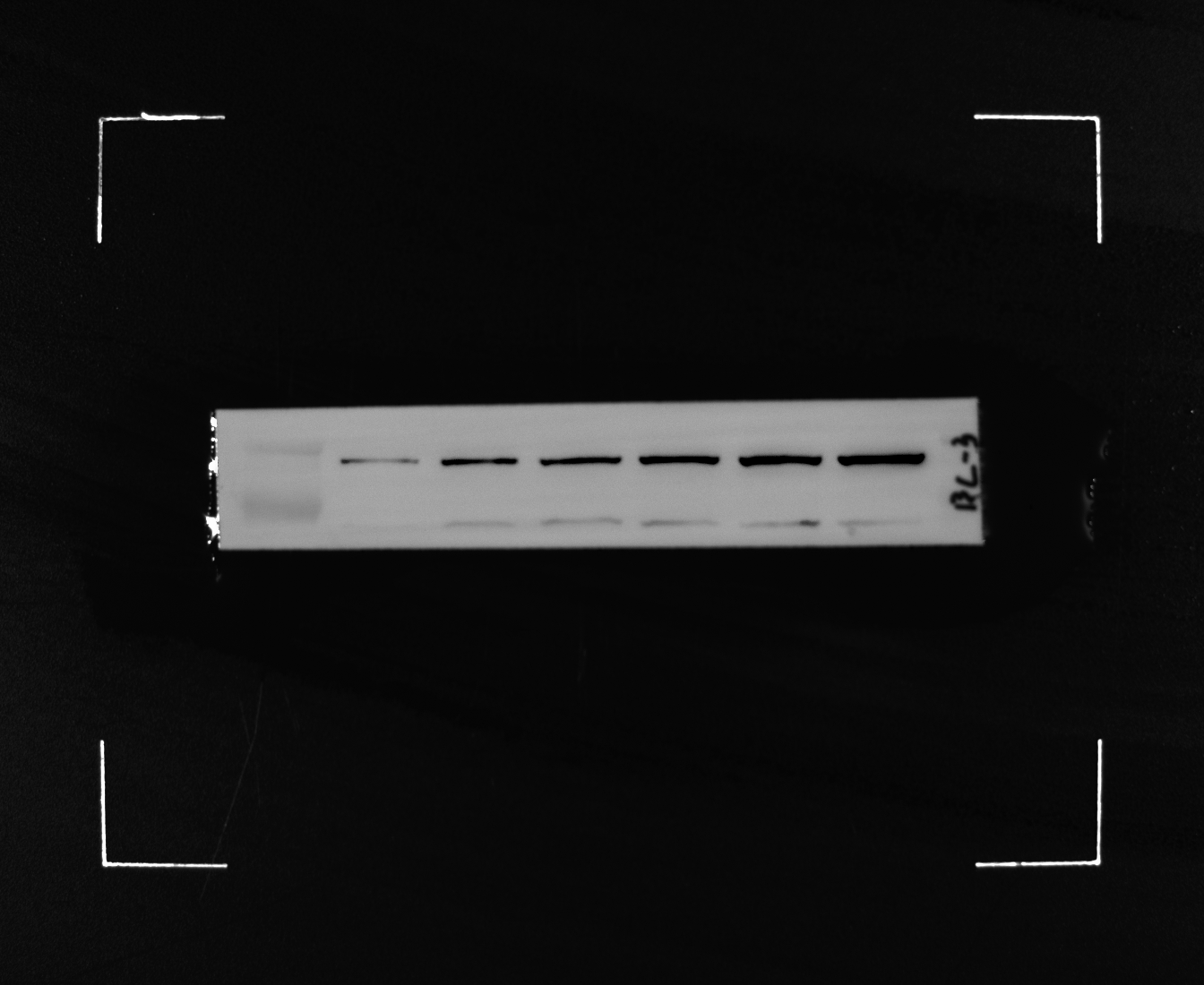

Supplement: Supplementary file 1 [file molecules-28-03842-s001.zip › WB original picture/SDF-1 CXCR4 a┬-catenin p-GSK3a┬/a┬-catenin-3_220728_112026_00.01.000_0_24210.tif]
